# Development and Application of the CRISPR‐dcas13d‐eIF4G Translational Regulatory System to Inhibit Ferroptosis in Calcium Oxalate Crystal‐Induced Kidney Injury

**DOI:** 10.1002/advs.202309234

**Published:** 2024-02-21

**Authors:** Ziqi He, Chao Song, Sheng Li, Caitao Dong, Wenbiao Liao, Yunhe Xiong, Sixing Yang, Yuchen Liu

**Affiliations:** ^1^ Department of Urology Renmin Hospital of Wuhan University Wuhan Hubei Province 430060 P. R. China; ^2^ Shenzhen Institute of Translational Medicine Shenzhen Second People's Hospital The First Affiliated Hospital of Shenzhen University Health Science Center Shenzhen University Shenzhen Guangdong Province 518035 P. R. China; ^3^ Department of Urology Zhongnan Hospital of Wuhan University Wuhan Hubei Province 430071 P. R. China; ^4^ Department of Biological Repositories Tumor Precision Diagnosis and Treatment Technology and Translational Medicine Hubei Engineering Research Center Zhongnan Hospital of Wuhan University Wuhan 430071 P. R. China

**Keywords:** CRISPR‐dCas13d‐eIF4G, ferroptosis Inhibition, kidney stone disease model, mRNA translation regulation, targeted protein expression

## Abstract

The CRISPR‐Cas system, initially for DNA‐level gene editing and transcription regulation, has expanded to RNA targeting with the Cas13d family, notably the RfxCas13d. This advancement allows for mRNA targeting with high specificity, particularly after catalytic inactivation, broadening the exploration of translation regulation. This study introduces a CRISPR‐dCas13d‐eIF4G fusion module, combining dCas13d with the eIF4G translation regulatory element, enhancing target mRNA translation levels. This module, using specially designed sgRNAs, selectively boosts protein translation in targeted tissue cells without altering transcription, leading to notable protein expression upregulation. This system is applied to a kidney stone disease model, focusing on ferroptosis‐linked GPX4 gene regulation. By targeting GPX4 with sgRNAs, its protein expression is upregulated in human renal cells and mouse kidney tissue, countering ferroptosis and resisting calcium oxalate‐induced cell damage, hence mitigating stone formation. This study evidences the CRISPR‐dCas13d‐eIF4G system's efficacy in eukaryotic cells, presenting a novel protein translation research approach and potential kidney stone disease treatment advancements.

## Graphical abstract

By combining CRISPR‐dcas13d's RNA‐targeting with eIF4G fusion protein's translation initiation regulation, a CRISPR‐dcas13d‐eIF4G translational regulatory system is used to modulate gene translation levels in mammalian cells. Using this platform to upregulate GPX4 translation significantly inhibits oxidative damage and ferroptosis in a crystal‐induced cell injury model. Additionally, it reduces crystal deposition in a kidney stone mouse model, thus suppressing stone formation.

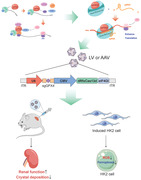

## 1 Introduction

Engineered CRISPR‐Cas systems are now widely used in gene editing and transcriptional regulation, with classical CRISPR‐Cas9 being particularly useful for editing specific gene loci in DNA and correcting mutations in disease‐causing genes.^[^ ^1^ ^]^ However, there has been limited development and research into simple and editable tools for RNA‐level editing, and methods for directly studying the functional roles of specific RNA sites and their engineering are quite scarce. Researchers have discovered that the Type VI CRISPR‐Cas system included a programmable single‐effector RNA‐guided enzyme called Cas13,^[^ ^2^ ^]^ which can target and guide RNA rather than DNA. The catalytically inactive version of Cas13 (dCas13) can be used for RNA editing work.^[^ ^2^, ^3^ ^]^ Cas13 is a distinctive single‐effector enzyme family composed of four different subtypes (Cas13a, Cas13b, Cas13c, and Cas13d) of RNA‐guided ribonucleases. Besides sharing two structurally conserved R‐X4‐6‐H HEPN domains, there is no significant sequence similarity among the four subtypes.^[^ ^4^ ^]^ Among them, Cas13d (930 aa) is currently the smallest subtype of CRISPR‐Cas13, ≈20–30% smaller than other subtypes, making it more suitable for packaging in a single viral vector. Engineering fusion tools related to CRISPR‐Cas13d can be packaged into a single viral vector, showing excellent transformation and application potential. Cas13d cannot cleave single‐stranded DNA or double‐stranded DNA corresponding to the target single‐stranded RNA, indicating that Cas13d is an RNA‐specific ribonuclease. Testing its cutting efficiency at different temperatures revealed that Cas13d exhibits high enzymatic activity within the range of 21–42 °C, suitable for most prokaryotic and eukaryotic hosts. This makes CRISPR‐Cas13d‐related engineering fusion tools highly promising for applications in mammalian cells. Silvana and others discovered a highly active Cas13d family ribonuclease effector (RfxCas13d) in mammalian cells through analysis of prokaryotic genomic and metagenomic sequences from the Ruminococcus flavefaciens xpd3002. Subsequent research found that RfxCas13d‐mediated RNA knockdown showed high cutting efficiency and target specificity for different endogenous transcripts, without affecting endogenous genome transcription, compared to traditional RNA interference techniques.^[^ ^5^ ^]^ At the same time, Silvana and colleagues found that reduced expression of the target gene depended on the catalytic activity of the HEPN domain in RfxCas13d. Wild‐type RfxCas13d can effectively mediate the knockdown of target mRNA, but the catalytically inactive RfxCas13d (dRfxCas13d) does not significantly affect the expression levels of target mRNA and its protein. This suggests that dRfxCas13d can efficiently target coding regions of mRNA without interfering with transcription and translation processes, providing many possibilities for the Cas13d family in RNA editing and protein translation regulation research.^[^ ^5^ ^]^ The discovery of CRISPR‐Cas13d and the development of engineered fusion tools like dRfxCas13d have expanded the genome editing toolbox from the DNA level to the RNA level, offering more possibilities for various gene engineering techniques. However, a single dRfxCas13d engineering fusion unit alone cannot regulate mRNA translation, which is the final step in gene expression and directly determines the strength of gene expression. Although gene overexpression vectors or CRISPR‐mediated transcriptional activation can significantly increase mRNA expression levels for many genes, mRNA and protein expression levels are not always proportional. Protein expression levels still depend on translation efficiency, making it crucial to seek functional elements for effective translation activation.

Eukaryotic translation initiation factor 4G (eIF4G) is an important modular fusion protein that forms the translation initiation complex (eIF4F) by binding with RNA helicase (eIF4A) and cap‐binding protein (eIF4E). It serves as a critical target for translation regulation in many biological processes. eIF4G, as an essential protein for translation initiation, mainly exerts its function through three functional regions: the N‐terminal, the central region, and the C‐terminal. The N‐terminal of eIF4G contains binding sites for polyA binding protein and eIF4E.^[^ ^6^, ^7^ ^]^ The central region of eIF4G is the most highly conserved region and includes binding domains for eIF3, eIF4A, and mRNA recognition motifs.^[^ ^8^, ^9^ ^]^ The C‐terminal of eIF4G contains another binding domain for eIF4A and a kinase‐binding domain that activates eIF4E through phosphorylation.^[^ ^10^, ^11^ ^]^ eIF4G, through these primary functional regions, binds to eIF4E, eIF4A, and eIF3 to form the minimal structure supporting translation initiation, which is the core functional region of the translation initiation complex.^[^ ^12^, ^13^ ^]^ The eIF4G can simultaneously bind eIF3 and eIF4E, and this complex is considered a molecular bridge linking the 5′ end of mRNA and the ribosomal subunit, recruiting the 40S ribosomal subunit to the cap structure at the 5′ end of mRNA. The 40S ribosomal subunit, together with Met‐tRNA and eukaryotic translation initiation factors, forms the 43S preinitiation complex, moves along the 5′‐UTR until it encounters the first AUG codon, and then stops. It continues to recruit the 60S subunit to form the 80S ribosome and initiate peptide synthesis. Therefore, eIF4G is considered a crucial target for translation regulation.^[^ ^14^, ^15^ ^]^ There are two less conserved isoforms of eIF4G, eIF4GI and eIF4GII, both of which can form a functional eIF4F complex with eIF4E, eIF4A, and eIF3.^[^ ^16^ ^]^ However, in mammalian cells, eIF4GI is the predominant form of eIF4G, accounting for ≈85% of the total eIF4F complex.^[^ ^17^ ^]^ Therefore, eIF4GI fusion proteins can improve translation by enhancing ribosome recruitment without increasing mRNA levels and are operable in mammalian cells.

Because there are few technologies and tool platforms developed to activate mRNA translation, the eIF4GI fusion protein, which is crucial for translation enhancement, may be used to create new engineered elements for activating translation in mammalian cells. In our study, we designed and developed a translation activation tool based on CRISPR‐dRfxCas13d targeting specific mRNA in mammalian cells. By fusing the catalytically inactive RfxCas13d with eIF4GI and guiding it with specific sgRNAs, we aimed to stimulate the translation of mammalian protein‐coding genes. CRISPR‐dRfxCas13d‐eIF4G could effectively manipulate the translation activation of various endogenous genes with negligible off‐target challenges in mammalian cells. The advantage of the compact size of CRISPR‐dCas13d‐eIF4GI allows the fusion protein constructs to be packaged into a single lentivirus (LV) or adeno‐associated virus (AAV) vector, facilitating their packaging and delivery when used for molecular therapeutic strategies.

Furthermore, to enhance the efficiency and expand the applicability of the CRISPR‐dCas13d‐eIF4G translation regulation system in different scenarios, we applied this translation control system to a kidney stone disease model. Kidney stones are one of the most common urological diseases, characterized by a high incidence, high recurrence rate, and high treatment cost.^[^ ^18^, ^19^ ^]^ Among kidney stones, calcium oxalate (CaOx) is the most common type, accounting for 65.9% of all cases.^[^ ^20^ ^]^ Supersaturation of CaOx crystals in urine is a critical risk factor for stone formation.^[^ ^21^ ^]^ Therefore, we used a CaOx crystal‐cell injury model to simulate the crystal‐cell injury that occurs during kidney stone formation. Our previous research revealed that an increase in ferroptosis levels could exacerbate the severity of crystal‐cell injury and lead to increased crystal deposition in kidney tissue, and showed that GPX4 is a key inhibitory gene in ferroptosis.^[^ ^22^ ^]^ Thus, we selected GPX4 as our target gene and designed corresponding sgRNA sequences for its mRNA. Through the specific recognition of the GPX4 mRNA sequence in HK‐2 cells by the combined action of dRfxCas13d and sgRNA, the presence of the fusion group eIF4GI increased the translation level of GPX4 mRNA, mediating a significant upregulation of GPX4 protein expression in HK‐2 cells. This, in turn, helped to resist cell crystal injury induced by CaOx crystals, while simultaneously reducing the levels of ferroptosis and reactive oxygen species (ROS) in HK‐2 cells.

It is evident that the CRISPR‐dRfxCas13d‐eIF4G translation activation system holds great promise for biological applications. This study will provide a powerful tool platform for specific mRNA translation enhancement in mammalian cells and offer a novel molecular therapeutic approach for kidney stone treatment based on targeted RNA manipulation.

## 2 Results

### 2.1 Identification of Essential Proteins of Ferroptosis by Proteomic 4D‐LFQ

**Figure 1:**
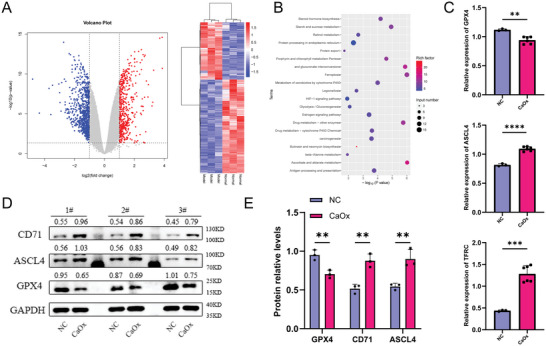
Identification of essential proteins of ferroptosis by 4D‐LFQ proteomics technology. A) Volcano plot (left) and heatmap plot (right) for DEGs between CaOx crystal‐induced cell injury models and normal controls. B) KEGG term analysis of DEGs in pathway analysis. C) Protein expression of GPX4, ASCL4, and TFRC (also called CD71) in HK‐2 cells was determined by proteomic 4D‐LFQ. D) Western blotting results show the protein expression levels of GPX4, ASCL4, and CD71 for the two groups and the bar graph (*n* = 3). E) shows the relative protein levels.

A total of 41 296 proteins were identified, of which 492 were significantly upregulated and 805 were downregulated (**Figure** **1**A; Figure S12A, Supporting Information). The results of GO and KEGG enrichment analyses revealed that most differentially expressed genes (DEGs) were associated with unfolded protein binding, xenobiotic metabolic process, xenobiotic glucuronidation, and other processes (Figure 1B; Figure S12B, Supporting Information). The ferroptosis pathway, which was significantly associated with differentially expressed proteins, was the focus of our attention. The hub genes of the ferroptosis pathway (MAP1LC3B2, TFRC, FTH1, PRNP, PCBP2, ACSL4, and GPX4) were differentially expressed in the model group (Figure 1C; Figure S12C,D, Supporting Information). We also validated the protein expression of TFRC, FTH1, PRNP, PCBP2, ACSL4, and GPX4 using a western blot (Figure 1D,E; Figure S12E, Supporting Information). And we selected GPX4, ACSL4, and TFRC (also called CD71) as the differentially expressed proteins to be validated in this study, considering both lipid peroxidation and iron ion overload.

### 2.2 Design and Verification of a Dual‐Vector CRISPR‐dRfxCas13d‐eIF4G Translational Regulatory Tool

**Figure 2:**
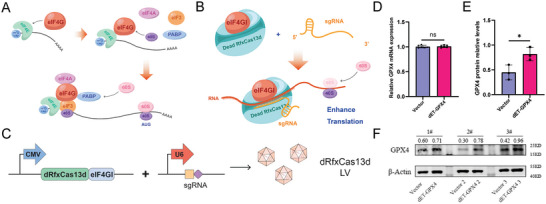
Design and construction of the dual‐vector CRISPR‐dRfxCas13d‐eIF4G translational regulatory tool targeting GPX4. A) The eIF4G combines with eIF4E, eIF4A, polyA binding protein, and eIF3 to form a minimal structure that supports translation initiation through the central functional region, which is the core functional region of the translation initiation complex. The 40S and 60S ribosomal subunits are recruited to become involved in peptide synthesis. B) The schematic design of the dual‐vector CRISPR‐dRfxCas13d‐eIF4GI. The type I isoform of eIF4G, eIF4GI, was selected to fuse with dead RfxCas13d. Then the CRISPR‐dRfxCas13d‐eIF4G tool targets sequences that bind specific RNAs and increases the translation level of their corresponding proteins, guided by sgRNAs. C) The dRfxCas13d‐eIF4GI fusion protein and sgRNA complementary DNA sequence of related genes were cloned into plasmids containing the CMV promoter and U6 promoter, respectively, for packaging into lentiviral vectors. D) qRT‐PCR analysis verified the mRNA expression level of GPX4 after constructing the CRISPR‐dRfxCas13d‐eIF4G tool targeting GPX4 (dET‐GPX4). E,F) Western blotting results verified the protein expression level of GPX4 after constructing the CRISPR‐dRfxCas13d‐eIF4G tool targeting GPX4 (dET‐GPX4) (*n* = 3). Data are presented as the means ± SEM from three independent experiments. ^*^ *p* < 0.05, ^**^ *p* < 0.01, ^***^ *p* < 0.001, and ^****^ *p* < 0.0001 versus the Vector group, and ns represents *p* > 0.05 versus the Vector group.

The eIF4G is a critical target for translational regulation in many biological processes and is essentially a modular fusion protein that forms the translation initiation complex (eIF4F) by binding RNA helicase (eIF4A) and cap‐binding protein (eIF4E). The complex is thought to be a molecular bridge between the 5′ end of the mRNA and the ribosomal subunit binding, capable of recruiting the 40S ribosomal subunit to the cap structure at the mRNA 5′ end. The 40S ribosomal subunit continues to recruit the 60S subunit to start peptide synthesis, which is an important step in the initiation of translation (**Figure** **2**A,B). Based on the above mechanism, we selected catalytically inactivated RfxCas13d (dRfxCas13d) as an RNA targeting element, and pSP4GI (the full‐length eIF4GI cDNA clone DKFZp762O191Q3, accession no. AL120751) as a translation function‐enhancing element,^[^ ^14^ ^]^ where pSP4GI was fused to the carboxy‐terminal of dRfxCas13d to form the translational regulatory structure domain. At the same time, the sequence of sgRNA was designed through the online design website https://cas13design.nygenome.org. The dRfxCas13d‐eIF4GI fusion protein and sgRNA drove the vector through the CMV promoter and U6 promoter, respectively. Silvana et al. reported that the RNA‐targeting specificity and cleavage activity of RfxCas13d was stronger when it was localized in the nucleus than in the cytoplasm, but the translation process in our study mainly occurred in the cytoplasm. Therefore, we divided the translational regulatory tools into the nuclear entry (NLS) and cytoplasmic (no NLS) groups (Figure 2C; Figure S13A, Supporting Information) by inserting or not inserting the NLS sequence at both ends of the dRfxCas13d‐eIF4G fusion protein, and the vectors of both groups and the sgRNA vector (MOI 50:50) were constructed as LV tools and inserted into the genome of HK‐2 cells. After stable transfection and screening, mRNA and protein levels of GPX4 were detected by RT‐qPCR and WB methods, respectively. The mRNA expression levels of GPX4 in both groups were not differentially altered compared with the negative control group. In contrast, the protein levels of GPX4 in the cytoplasmic (no NLS) group were significantly upregulated (Figure 2D–F), but no significant changes were observed in the nuclear entry (NLS) group (Figure S13B,C, Supporting Information). The above results allowed us to determine that translational regulation would mainly occur in the cytoplasm and that the dual vector tool could work stably in HK‐2 cells and increase the translation level of the target gene GPX4. Therefore, we selected the cytoplasmic (no NLS) dRfxCas13d‐eIF4GI translational regulatory tool for subsequent experiments.

### 2.3 Application of the Dual‐Vector CRISPR‐dRfxCas13d‐eIF4G Translational Regulatory Tool in the CaOx Crystal‐Induced Cell Injury Model

**Figure 3:**
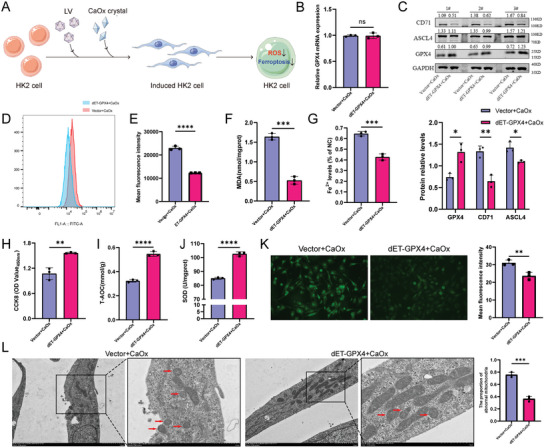
Application of the dual‐vector CRISPR‐dRfxCas13d‐eIF4G translational regulatory tool in a CaOx crystal‐induced cell injury model. A) The lentiviral plasmids with the dual‐vector CRISPR‐dRfxCas13d‐eIF4G translational regulatory tool (dET‐GPX4) or vector were transfected into HK2 cells, and HK‐2 cells were treated with CaOx intervention solution (2 mm) for 24 h. B) The mRNA expression of GPX4 in HK‐2 cells was determined using qRT‐PCR analysis for the two groups. C) Western blotting results show the protein expression levels of GPX4, ASCL4, and CD71 for the two groups and the bar graph shows the relative protein levels. D) The data from flow cytometric analysis shows the degree of lipid peroxidation in the two groups and the bar graph E) shows the mean fluorescence intensity. Several indicators of ferroptosis, including MDA content (F) and Fe^2+^ content (G), were measured. H) CCK‐8 assays were used to detect cell viability. Several indicators of oxidative and anti‐oxidative status, including T‐AOC content (I) and SOD content (J) were measured. K) Images were taken under a dark field (magnification, x200). Brighter green indicates higher levels of cellular ROS and the bar graph shows the mean fluorescence intensity. L) The mitochondrial morphology alterations associated with ferroptosis were observed using transmission electron microscopy (scale bars = 2 µm or 1 µm). Red arrows represent abnormal mitochondria (solid and broken mitochondria) and the bar graph shows the ratio of the number of abnormal mitochondria to the total number of mitochondria in the current field. Data are presented as the means ± SEM from three independent experiments (*n* = 3). One set of representative images from three independent experiments is shown. ^*^ *p* < 0.05, ^**^ *p* < 0.01, ^***^ *p* < 0.001, and ^****^ *p* < 0.0001 versus the Vector+CaOx group.

The above experiments verified the effectiveness of the dual‐vector translational regulatory tool in normal cultured HK‐2 cells. We next constructed a crystal‐induced cell injury model using CaOx crystals in HK‐2 cells after stable transfection of the tool vector and found that the stable function of the dual‐vector CRISPR‐dRfxCas13d‐eIF4G translational regulatory tool was not affected after crystal modeling. Meanwhile, GPX4, an essential suppressor gene of ferroptosis, inhibited ferroptosis by downregulating lipid peroxidation levels.^[^ ^23^ ^]^ We found a possible reciprocal regulatory relationship between GPX4 and ferroptosis agonist genes (ACSL4 and CD71) in the CaOx crystal‐induced cell injury model by 4D‐LFQ proteomics technology (Figure S14, Supporting Information). After observing that the translation level of GPX4 was upregulated by the translational regulatory tool but the transcription level of GPX4 was unchanged (**Figure** **3**B). We found that the GPX4 protein level was upregulated significantly as verified by WB. And the protein level of CD71 was significantly downregulated and the ASCL4 protein expression was slightly decreased in the dET‐GPX4+CaOx group (Figure 3C). These results demonstrated that the dual‐vector CRISPR‐dRfxCas13d‐eIF4G translational regulatory tool could indirectly regulate the protein expression levels of ACSL4 and TFRC, which may interact with GPX4 after upregulating the translation level of GPX4, a key inhibitor of ferroptosis. The detection of ferroptosis‐related indices and antioxidant capacity indicated a significant reduction in the intracellular levels of lipid peroxidation and its metabolite MDA as well as Fe^2+^ (Figure 3D–G), and also some reversion in cellular viability (Figure 3H). In contrast, intracellular levels of both T‐AOC and SOD, the cellular antioxidant indicators, were significantly higher (Figure 3I,J), along with intracellular levels of ROS after upregulating GPX4 translation by the translational regulatory tool (Figure 3K). When ferroptosis occurs, the morphology of mitochondria will change, such as decreased volume, increased mitochondrial membrane density, decreased mitochondrial cristae mitochondrial, or membrane rupture, respectively.^[^ ^24^ ^]^ Mitochondrial morphology was observed by transmission electron microscopy, and it was found that the number of mitochondria was significantly increased after GPX4 translation levels were upregulated by the translational regulatory tool compared to the negative control vector group, and the number of solid and broken mitochondria was significantly reduced compared to the negative control vector group (Figure 3L). The above results demonstrated that the dual‐vector CRISPR‐dRfxCas13d‐eIF4G translational regulatory tool could function stably in the CaOx crystal‐induced cell injury model. This tool could reduce the protein expression of ferroptosis‐related agonist genes, inhibit the level of lipid metabolism and the intracellular accumulation of Fe^2+^, enhance the cellular antioxidant capacity, improve mitochondrial function, and increase cellular viability. Thus, it could inhibit the level of ferroptosis and the extent of crystal‐induced injury in the CaOx crystal‐induced cell injury model, play a favorable cytoprotective role, and inhibit the CaOx crystal‐induced injury.

### 2.4 Design and Specificity of the Single‐Vector CRISPR‐dRfxCas13d‐eIF4G Translational Regulatory Tool

**Figure 4:**
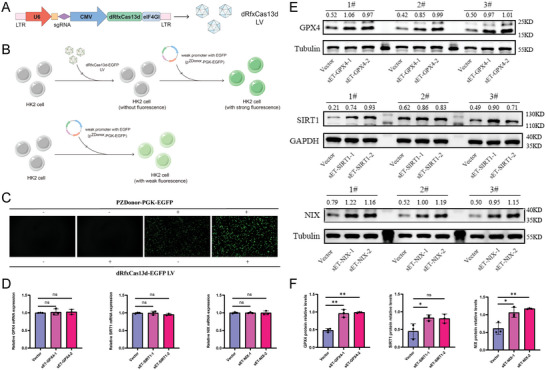
Design, construction, and evaluation of the single‐vector CRISPR‐dRfxCas13d‐eIF4G translational regulatory tool in the CaOx crystal‐induced cell injury model. A) The single‐vector CRISPR‐dRfxCas13d‐eIF4GI and sgRNA complementary to the DNA sequence of related genes were cloned into the plasmids containing both the CMV promoter and U6 promoter for packaging into lentiviral vectors. B) Flow diagram for verifying that the translational regulatory tool targets the weak promoter‐driven EGFP. C) The plasmids containing the weak promoter with EGFP (pZDonor‐PGK‐EGFP) were transfected into HK‐2 cells with or without the CRISPR‐dRfxCas13d‐eIF4G translational regulatory tool. Images were taken under a dark field (magnification, ×100). Brighter green indicates higher translation levels of the weak promoter‐driven EGFP. D) The lentiviral plasmids with the single‐vector CRISPR‐dRfxCas13d‐eIF4G translational regulatory tool (sET‐GPX4, sET‐SIRT1, and sET‐NIX) or vector were transfected into HK2 cells. The mRNA expression of GPX4, SIRT1, and NIX in HK‐2 cells was determined using qRT‐PCR analysis. E) Western blotting results show the protein expression levels of GPX4, SIRT1, and NIX for the three groups, and F) the bar graph shows protein quantification. Data are presented as the means ± SEM from three independent experiments (*n* = 3). ^*^ *p* < 0.05, ^**^ *p* < 0.01, ^***^ *p* < 0.001, and ^****^ *p* < 0.0001 versus the Vector group, and ns represents *p* > 0.05 versus the Vector group.

Although the dual‐vector CRISPR‐dRfxCas13d‐eIF4G translational regulatory tool was shown in our hands to be stably expressed and to function in normal culture environments and relevant disease models, this was no guarantee that the dRfxCas13d‐eIF4GI fusion protein vector and the sgRNA vector could be stably expressed in all cells simultaneously. In order to verify the subcellular localization of the single vector CRISPR‐dRfxCas13d‐eIF4G translation enhancement tool and its consistency with the dual vector CRISPR‐dRfxCas13d‐eIF4G translation enhancement tool, we fused the Cherry fluorescent label onto the plasmid skeleton tail of the nuclear entry (NLS) and the cytoplasmic (no NLS) dRfxCas13d‐eIF4G translation regulation tools, and packaged all the fusion protein tools into a single LV vector. The sgRNA was designed for GPX4. After transfecting HK‐2 cells separately, we observed fluorescence localization in the two sets of translational regulatory tools and they could accurately locate in the nucleus or cytoplasm, respectively, based on whether they carried NLS tags. The mRNA and protein content of GPX4 were measured by RT‐qPCR and WB, and stable transfection with nuclear entry (NLS) and cytoplasmic (no NLS) dRfxCas13d‐eIF4GI‐GPX4 was compared with the negative control, respectively. There was no significant difference in the mRNA levels of GPX4 between the two groups, while after stable transfection with nuclear entry (NLS) dRfxCas13d‐eIF4GI‐GPX4, the protein content of GPX4 showed no significant change compared to the negative control. However, after stable transfection with cytoplasmic (no NLS) dRfxCas13d‐eIF4GI‐GPX4, the protein content of GPX4 was significantly upregulated compared to the negative control. Based on the above results, the single‐carrier CRISPR‐dRfxCas13d‐eIF4G translation enhancement tool and the dual‐carrier CRISPR‐dRfxCas13d‐eIF4G translation enhancement tool were demonstrated to show consistent subcellular localization in the working area, both of which could work effectively in the cytoplasm (Figure S15, Supporting Information). Then, we sought to construct a single‐vector LV tool to achieve a more stable expression of the CRISPR‐dRfxCas13d‐eIF4G translational regulatory tool and thus improve its efficiency. By deleting the non‐essential tool elements, all the essential tool elements were placed in a single LV vector, with the sgRNA expression and dRfxCas13d‐eIF4G fusion protein expression driven by the U6 promoter and CMV promoter, respectively (**Figure** **4**A).

EGFP is an exogenous fluorescent protein that is not expressed in mammalian cells. A weak promoter‐driven EGFP (pZDonor‐PGK‐EGFP) plasmid was constructed and transfected into HK‐2 cells with negative control plasmids. After 8 h, it was observed that HK‐2 cells transfected with the weak promoter‐driven EGFP plasmids showed weak fluorescence expression, while the negative control group did not show any fluorescence expression. To verify that the translational regulatory tool could only bind mRNAs that recognized the target gene, we designed the corresponding sgRNA sequence for the weak promoter‐driven EGFP. After stable transfection and expression of dRfxCas13d‐eIF4GI‐EGFP in HK‐2 cells, the negative control plasmid and pZDonor PGK‐EGFP plasmid were transfected separately (Figure 4B). We observed the fluorescence expression intensity of the two groups of cells after 8 h of transfection and found that the fluorescence intensity of HK‐2 cells transfected with pZDonor PGK‐EGFP plasmid was significantly enhanced after stable expression of dRfxCas13d‐eIF4GI‐EGFP, while HK‐2 cells transfected with the negative plasmid after stable expression of dRfxCas13d eIF4GI‐EGFP did not show any fluorescence expression (Figure 4C). We then stably transfected and expressed the dRfxCas13d‐eIF4GI‐EGFP and dRfxCas13d‐eIF4GI‐vectors in HK‐2 cells, and transfected the two groups of HK‐2 cells using the pZDonor PGK‐EGFP plasmid for protein mass spectrometry analysis. Only EGFP showed significant differential expression in the two groups of cell samples, while other endogenous proteins did not show significant differential transformation (Figure S16 and Table S4, Supporting Information). Therefore, we have shown that the dRfxCas13d‐eIF4G translation enhancement tool had good specificity with almost no off‐target effects.

### 2.5 General Applicability Validation of the Single‐Vector CRISPR‐dRfxCas13d‐eIF4G Translational Regulatory Tool

To further investigate the translational regulatory capacity of the single‐vector CRISPR‐dRfxCas13d‐eIF4G translational regulatory tool in mammalian cells, we selected GPX4 and two other endogenous genes, namely NIX and SIRT1. NIX plays a vital role in the regulation of mitochondrial autophagy, while SIRT1, a classical longevity gene, plays an antioxidant role in various kidney‐related diseases. After designing two sgRNAs for the endogenous genes, a single vector CRISPR‐dRfxCas13d‐eIF4G translation regulation tool was constructed. After stable expression, mRNA and protein expression were detected using qRT‐PCR and WB, respectively. The results showed that the mRNA levels of GPX4, NIX, and SIRT1 were not differentially changed compared with the negative control group (Figure 4D). Still, protein levels of GPX4, NIX, and SIRT1 were significantly and differentially changed compared with the negative control group (Figure 4E,F). These results suggested that the single vector CRISPR‐dRfxCas13d‐eIF4G translational regulatory tool had universal applicability and excellent prospects for development and application.

### 2.6 Efficiency Evaluation of the Single‐Vector CRISPR‐dRfxCas13d‐eIF4G Translational Regulatory Tool

**Figure 5:**
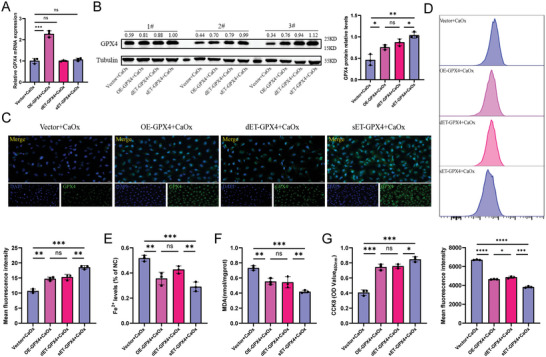
Verification of the single‐vector CRISPR‐dRfxCas13d‐eIF4G translational regulatory tool (sET‐GPX4). A) The bar graph shows mRNA relative levels of GPX4 for four groups. B) Western blotting results show the protein expression levels of GPX4 for the four groups and the bar graph shows protein quantification. C) Immunofluorescence assays were performed to further determine the expression of GPX4 (magnification, ×200). The bar graph shows relative protein levels. D) Data from flow cytometric analysis shows the degree of lipid peroxidation in four groups and the bar graph shows the mean fluorescence intensity. Several indicators of ferroptosis, including Fe^2+^ content (E) and MDA content (F), were measured. G) The bar graph shows cell viability for the four groups. Data are presented as the means ± SEM from three independent experiments. One set of representative images from three independent experiments is shown. Data are presented as the means ± SEM from three independent experiments (n = 3). ^*^ *p* < 0.05, ^**^ *p* < 0.01, ^***^ *p* < 0.001, and ^****^ *p* < 0.0001, and ns represents no significant difference.

After HK‐2 cells stably expressed the single‐vector CRISPR‐dRfxCas13d‐eIF4G translational regulatory tool, we compared their gene regulation efficiency with the conventional LV‐mediated gene overexpression tool and the dual‐vector CRISPR‐dRfxCas13d‐eIF4G translational regulatory tool groups. The mRNA and protein levels of GPX4 were detected by qRT‐PCR and WB methods, respectively. It was found that the mRNA levels of GPX4 were significantly higher in cells after stable expression of the conventional LV overexpression tool compared with the two types of CRISPR translational regulatory tools. Still, there was no significant difference in GPX4 protein expression levels after stable expression of the conventional LV overexpression tool and the dual‐vector CRISPR‐dRfxCas13d‐eIF4G translational regulatory tool (**Figure** **5**A). In contrast, the GPX4 protein expression levels were significantly higher in the single‐vector group compared with the other two groups (Figure 5B). Meanwhile, the previous results were reconfirmed by immunofluorescence (Figure 5C). The cellular ferroptosis‐related indices, antioxidant ability, and cellular viability of the three groups were further examined, and there were no differential changes in these indices between the two groups after stable expression of the conventional LV overexpression tool and the dual‐vector CRISPR‐dRfxCas13d‐eIF4G translational regulatory tool. In addition, expression of the single‐vector CRISPR‐dRfxCas13d‐eIF4G translational regulatory tool resulted in significantly lower intracellular levels of the lipid peroxidation metabolites MDA and Fe^2+^ (Figure 5D–F) compared to the other two groups, as well as significantly higher cell viability (Figure 5G). These results indicated that the single‐vector CRISPR‐dRfxCas13d‐eIF4G translational regulatory tool may function as a more efficient translational level activator against the target gene GPX4 through more stable intracellular expression. Compared with the conventional LV overexpression tool, the single‐vector CRISPR‐dRfxCas13d‐eIF4G translational regulatory tool had higher efficiency in upregulating the protein level of target gene GPX4, and the inhibitory effect on ferroptosis and cell protection was significantly improved for both. Therefore, we selected the single‐vector CRISPR‐dRfxCas13d‐eIF4G translational regulatory tool for the subsequent study.

### 2.7 Application of the Single‐Vector CRISPR‐dRfxCas13d‐eIF4G Translational Regulatory Tool in the Mouse Kidney Stone Model

**Figure 6:**
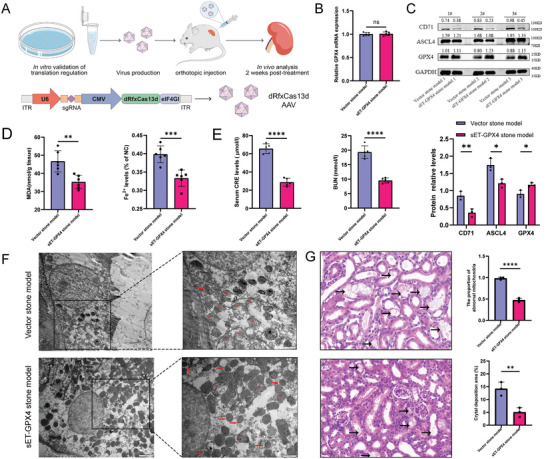
Application of the single‐vector CRISPR‐dRfxCas13d‐eIF4G translational regulatory tool in the mouse kidney stone model. A) The dRfxCas13d‐eIF4GI fusion protein and sgRNA complementary to the DNA sequence of related genes were inserted into the consent skeleton containing the CMV and U6 promoters to form the plasmid dRfxCas13d‐eIF4GI‐sgRNA for packaging into adeno‐associated viral vectors. Then the adeno‐associated viral plasmids were injected orthotopically into mice, and 80 mg kg^−1^ glyoxylic acid was injected i.p. into the mice every day for 14 days. B) The mRNA expression of GPX4 in HK‐2 cells was determined using qRT‐PCR analysis for the two groups. C) Western blotting results show the protein expression levels of GPX4, ASCL4, and CD71 for the two groups and the bar graph shows the relative protein levels (*n* = 3). D) Several indicators of ferroptosis, including MDA content and Fe^2+^content, were measured. E) Several indicators of kidney injury, including serum CRE content and BUN content, were measured (*n* = 6). F) The mitochondrial morphology alterations associated with ferroptosis were observed using transmission electron microscopy (scale bar = 2 or 1 µm). Red arrows represent solid mitochondria and red star marks represent broken mitochondria. The bar graph shows the ratio of the number of abnormal mitochondria to the total number of mitochondria in the current field. G) Crystal deposition was observed by calcium salt staining and H&E staining and the black arrows indicate crystals (not limited to these arrows) (magnification, ×400). The bar graph shows the area of crystal deposition in the current field. ^*^ *p* < 0.05, ^**^ *p* < 0.01, ^***^ *p* < 0.001, and ^****^ *p* < 0.0001 versus the vector stone model group.

Finally, to evaluate the efficiency of the single‐vector CRISPR‐dRfxCas13d‐eIF4G translational regulatory tool in vivo, we designed and constructed the single‐vector AAV translational regulatory tool following the above vector‐construction concept. After the orthotopic injection of AAV into the mouse kidney, mouse kidney stone modeling and sampling was performed after stable expression of the single‐vector AAV translational regulatory tool in kidneys (**Figure** **6**A). We were impressed to discover that the single‐vector AAV translational regulator tool could also be stably expressed and function in the kidneys of kidney stone model mice. It was confirmed through RT‐qPCR, WB, and immunohistochemistry that the single‐vector AAV translational regulatory tool could stably upregulate target gene GPX4 translation, significantly increase the corresponding protein expression level without affecting its mRNA transcript, and indirectly regulate the protein expression of GPX4‐interacting proteins ACSL4 and TFRC (Figure 6B,C; Figure S17, Supporting Information). In addition, the intracellular levels of lipid peroxidation metabolites MDA and Fe^2+^ were significantly lower in the target gene group compared with the negative control group (Figure 6D), and the renal function of the model mice recovered to some degree (Figure 6E). Projection electron microscopy was applied to detect mitochondrial morphological changes in the kidney tissue and showed that the target gene group possessed a significantly higher number of mitochondria and a significantly lower percentage of mitochondrial consolidation and fragmentation than the negative control group (Figure 6F). The experiment also revealed that the stable operation of the single‐vector AAV translational regulatory tool designed for the GPX4 gene could effectively alleviate kidney injury and diminish the extent of ferroptosis in the kidneys of the kidney stone mouse model. Finally, calcium salt staining and H&E staining showed that cavities were formed in the kidney tissues of the negative control group due to model damage, accompanied by loosening of the tissue structure and deposition of more calcium‐containing crystals in the cavities (Figure 6G). In contrast, the target gene group had significantly denser kidney tissue structure and fewer calcium‐containing crystals because of the protective effect of the stable expression and functioning of the translational regulatory tool, which directly reduced the deposition of calcium‐containing crystals in the kidneys of the model mice. In previous studies, we found that the deposition of calcium‐containing crystals in mouse kidneys could be inhibited by suppressing the level of ferroptosis in the kidneys of the kidney stone model.^[^ ^22^, ^25^ ^]^ In this study, we successfully demonstrated the effectiveness of the single‐vector CRISPR‐dRfxCas13d‐eIF4G translational regulatory tool in mammalian models in vivo, and have applied this gene tool for the first time to the best of our knowledge to treat kidney stones. Our results indicated that the CRISPR‐dRfxCas13d‐eIF4G translational regulatory tool possessed the potential for broader application, and further work on its development and design should be promising.

## 3 Discussion

The CRISPR‐Cas systems are primarily classified into Class 1 and Class 2 systems. Class 2 systems typically consist of a single large multi‐domain Cas protein, and their loci organization is simpler and more uniform than that of Class 1 systems, providing greater flexibility for modification.^[^ ^26^, ^27^ ^]^ They have been adapted into highly versatile gene engineering platforms and have been applied successfully in various fields, including gene editing, multicolor chromosome imaging, and molecular diagnostics, yielding significant achievements.^[^ ^1^, ^28^ ^]^ The Class 2 VI systems have been reported as CRISPR ribonucleases targeting RNA,^[^ ^2^, ^3^, ^5^, ^29^, ^30^, ^31^, ^32^ ^]^ with the CRISPR‐Cas13 system showing great potential for RNA manipulation.^[^ ^2^, ^3^, ^5^, ^33^ ^]^ Due to its efficient and specific RNA targeting capabilities, RNA manipulation platforms based on the CRISPR‐Cas13 system have been widely applied in RNA editing of yeast, plants, flies, zebrafish, and mammals.^[^ ^2^, ^5^, ^34^, ^35^, ^36^, ^37^ ^]^ Previous research has demonstrated the stable operation of RNA manipulation tools based on the CRISPR‐Cas13d system in animal models of relevant diseases.^[^ ^38^, ^39^, ^40^ ^]^ However, there has been little exploration of CRISPR systems for regulating gene expression at the translation level, which is not only a tool for studying fundamental biological phenomena but is also a potential pathway for new therapeutic strategies and biopharmaceutical production. In eukaryotic cells, eIF4GI fusion proteins can activate translation by enhancing ribosome recruitment without increasing mRNA levels. We developed a CRISPR‐dRfxCas13d‐eIF4GI translation control system that, upon introducing it into mammalian cells, specifically enhances the translation of target mRNA without altering transcription levels. This system stands out for its minimal off‐target effects and its ability to upregulate protein expression more effectively than similar tools, achieving higher protein levels without changing the genetic information of target cells. This efficiency and specificity make it a valuable tool for potential therapeutic applications and biopharmaceutical production.

In this study, we introduced the CRISPR‐dRfxCas13d‐eIF4G translation control system into HK‐2 cells and mouse kidney tissues using LV and AAV vectors. We applied this translation control system to a kidney stone disease model, making improvements and optimizations from the initial design. Our research team has long been involved in studying the mechanisms of kidney stone formation. Based on our preliminary research, we found that stimulation of HK‐2 cells with CaOx crystals can lead to a significant increase in intracellular lipid peroxidation levels and Fe^2+^ levels, further causing an increase in the level of ferroptosis. Conversely, after regulating the level of ferroptosis in the cell model with ferroptosis inhibitors, the degree of crystal‐induced cell damage significantly decreased, and the degree of crystal deposition within the cell tissue was significantly reduced.^[^ ^22^ ^]^ Therefore, we preliminarily confirmed the role of ferroptosis in promoting the formation of kidney stones. Further research showed that the overload of intracellular Fe^2+^ in the CaOx crystal‐mediated damage model may be due to the reduced expression level of FTH1. Further studies found that the protein expression of NCOA4 was increased in cells stimulated by CaOx crystals. And as an important positive regulator of autophagy, the increased expression of NCOA4 can cause autophagic degradation of FTH1, further leading to the activation of ferroptosis and exacerbating crystal deposition, ultimately promoting the formation of kidney stones.^[^ ^25^ ^]^ On the other hand, we found that HK‐2 cells stimulated by CaOx crystals can secrete exosomes highly expressing AMBRA1, which can induce autophagy‐dependent ferroptosis and induce oxidative damage to cells through the AMBRA1‐BECLIN1‐XCT axis.^[^ ^41^ ^]^ Our aforementioned preliminary research has proven that ferroptosis plays an important role in various types of crystal‐induced cell damage models and participates in the process of crystal deposition in tissues and the formation of kidney stones. Other researchers have also proposed that in HK‐2 cells stimulated by oxalate, the overexpression of the SOX4/EZH2 axis can activate ferroptosis by epigenetically suppressing the expression of SLC7A11, thus promoting oxidative damage and exacerbating crystal deposition in HK‐2 cells.^[^ ^42^ ^]^ Furthermore, in the CaOx crystal cell damage model, von Hippel‐Lindau tumor suppressor (VHL) mediates the proteasomal ubiquitination degradation of BICD2, further inhibiting STAT1 nuclear translocation, inhibiting IFNγ signal transduction, downregulating the expression of XCT mediated by IFNγ, ultimately reducing the cell's sensitivity to ferroptosis, thereby protecting cells from crystal‐induced damage and inhibiting the formation of kidney stones.^[^ ^43^ ^]^ Integrating the above research findings, it is evident that ferroptosis plays a crucial role in the formation of kidney stones.

Through 4D‐LFQ quantitative proteomics analysis of a CaOx kidney stone cell model, we found significant differences in the expression levels of key ferroptosis‐related proteins, including GPX4, TFRC, and ACSL4 in the modeling group compared to the control group. GPX4 is the only subunit of glutathione peroxidase that detoxifies lipid peroxides, playing a significant role in inhibiting lipid peroxidation and oxidative stress.^[^ ^23^, ^44^ ^]^ It is a crucial inhibitory gene for ferroptosis and served as the target gene for our subsequent validation experiments. Prior studies have reported that the RfxCas13d‐NLS fusion protein has superior target cleavage efficiency compared to the wild‐type RfxCas13d.^[^ ^5^ ^]^ However, unlike the transcription process that occurs in the cell nucleus, translation primarily takes place in the cytoplasm. Therefore, we constructed two classes of dRfxCas13d‐eIF4GI. One class is the nuclear entry dual vector. This class involves fusing eIF4GI to the C‐terminus of dRfxCas13d, followed by fusion of nuclear localization signals (NLS) at the N‐terminus and C‐terminus of the fusion protein complex to facilitate vector entry into the nucleus. The other class is the non‐nuclear dual vector (cytoplasmic dual vector), where eIF4GI is directly fused to the C‐terminus of dRfxCas13d without NLS signals. After stable transfection in HK‐2 cells, both the nuclear entry dual vector and the cytoplasmic dual vector can target either the cell nucleus or cytoplasm. However, the nuclear entry dual vector failed to function, while the cytoplasmic dual vector worked effectively. Therefore, we selected the CRISPR‐dRfxCas13d‐ eIF4GI cytoplasmic dual vector for subsequent experiments. Upon stable transfection with the CRISPR‐dRfxCas13d‐ eIF4GI dual vector for targeting GPX4 activation, we established a CaOx cell‐crystal damage model. It was observed that the CRISPR‐dRfxCas13d‐eIF4G translation control tool effectively increased GPX4 protein expression in HK‐2 cells (with no statistically significant change at the mRNA level) and suppressed ferroptosis and oxidative stress levels. Hence, it was evident that the CRISPR‐dRfxCas13d‐eIF4G translation enhancement tool could be stably expressed and effectively work in mammalian cell models of relevant diseases.

While dual vector systems commonly exhibit shortcomings in transfection and unstable expression efficiency, we improved the efficiency of the tool vectors further by optimizing and removing certain components (such as fluorescent tags). We integrated fusion functional elements and sgRNAs into single LV or AAV vectors. Subsequent research indicated that the mechanism of action of this single‐vector system was similar to that of the dual‐vector tool, allowing stable expression and high efficiency in HK‐2 cells. The single‐vector CRISPR‐dRfxCas13d‐eIF4G translation enhancement tool could efficiently upregulate various endogenous proteins (such as GPX4/SIRT1/NIX) and could also introduce exogenous proteins (e.g., EGFP) for protein expression in mammalian cells. In this study, we stably transfected dRfxCas13d‐eIF4GI‐EGFP and dRfxCas13d‐eIF4GI‐vector into HK‐2 cells, followed by separate transfections with EGFP guided by a weak promoter plasmid. Protein analysis using quantitative mass spectrometry showed no significant difference between the two groups, except for the increased expression of EGFP under the experimental conditions. This indicated that the single‐vector CRISPR‐dRfxCas13d‐eIF4G translation enhancement tool did not affect the expression of non‐targeted proteins and was not associated with significant off‐target effects.

In this study, we next constructed a kidney stone disease model by stably transfecting the single‐vector CRISPR‐dRfxCas13d‐eIF4G translation enhancement tool into HK‐2 cells and mouse kidney tissues. This tool significantly increased protein expression by upregulating GPX4 translation, effectively suppressing ferroptosis and oxidative stress levels in HK‐2 cells. It mitigated the extent of cell‐crystal damage, alleviated kidney function impairment, reduced crystal deposition in kidney tissues, and notably inhibited kidney stone formation. It was evident that the CRISPR‐dRfxCas13d‐eIF4G translation enhancement tool could be stably expressed in mammalian tissues to significantly increase the translation rate of target genes. Moreover, it worked efficiently in disease models, showing promising applications and development prospects.

In summary, our innovation built upon the potent editing capabilities of catalytically inactive RfxCas13d by fusing it with the eIF4GI protein. This fusion stimulated the cap‐dependent translation initiation process after the recognition and binding of target mRNA by dRfxCas13d. As a result, it enhanced the translation rate of target gene mRNA, all without altering the background DNA and mRNA levels. Our translation control tool achieved this by ribosome enrichment, leading to an increase in protein expression. This represented a novel approach for enhancing translation control within mammalian organisms. Traditional gene overexpression techniques involve introducing the full‐length sequence of the target gene from an external source, typically increasing the DNA background level to achieve gene overexpression. However, our CRISPR‐dRfxCas13d‐eIF4G translation control tool worked by directly regulating the translation initiation rate of endogenous target gene mRNA, raising the corresponding protein expression level without changing the background DNA and mRNA levels. This approach may significantly outperform traditional overexpression tools. Given that proteins serve as carriers of molecular function, it is important to recognize that increases in the transcription level of target genes often do not correspond linearly to increases in protein expression levels. Our translation control tool directly governed the initiation of protein translation, a feat that traditional overexpression techniques find challenging to achieve. Furthermore, protein translation level imbalances play a critical role in kidney‐related diseases such as diabetic nephropathy, renal cell carcinoma, polycystic kidney disease, and nephritis.^[^ ^45^ ^]^ Over 130 therapeutic proteins have already been employed, with more, including antibodies, in development.^[^ ^46^ ^]^ Because the initiation stage of translation represents a vital rate‐limiting step in translation control,^[^ ^47^ ^]^ our CRISPR‐dRfxCas13d‐eIF4G translation enhancement tool, which enhanced the translation initiation rate of the target RNA, has been successfully applied in the kidney stone disease model. It holds great potential in a wide range of related diseases characterized by protein translation level imbalances, offering a new perspective and a versatile tool platform for translation control research and the future treatment of protein translation‐related diseases.

## 4 Experimental Section

### Cell Culture and Model Establishment

HK‐2 cells were purchased from the National Collection of Authenticated Cell Cultures, cultured in a 37 °C, 5% CO_2_ environment, and maintained in DMEM/F12 medium supplemented with 10% fetal bovine serum. To establish a CaOx crystal damage model, HK‐2 cells were treated with a 1 mm CaOx intervention solution (CaOx added to DMEM/F12) for 24 h. For stable transfection experiments, HK‐2 cells were inoculated into a 6‐well plate one day before transfection. After reaching 50% confluency, the double‐carrier translation regulation system transfection reagent and LV virus mixture were configured at MOI 50:50, while the single‐carrier translation regulation system transfection reagent and LV mixture were configured at MOI 50, according to the manufacturer's instructions. After 8−12 h of transfection, the transfection was terminated by removing the transfection reagent. Stable cell lines were obtained by using DMEM/F12 medium containing puromycin (10 µg mL^−1^) for intermittent screening over 2 weeks. For transient transfection experiments, HK‐2 cells were inoculated into a 6‐well plate one day before transfection. After reaching 70–80% confluency, the cells were treated with Lipofectamine 2000 transfection reagent and plasmid mixture (1 µg µL^−1^) for 6 h, and then the transfection reagent was removed to terminate the transfection. The transient cell line was thus obtained.

### Animals

All animal experimental protocols were approved by Zhonghong Boyuan Biotechnology Co., Ltd (IACUC Issue No. 2 021 110 201). A total of 12 6‐week‐old male C57BL/6J mice (24–28 g) were purchased from Hubei Provincial Centers for Disease Control and Prevention. All mice were housed in specific pathogen‐free conditions with a steady humidity (40–70%) and temperature (22 ± 2 °C) barrier system under a 12‐h light‐dark cycle and provided with food and water ad libitum. The 12 mice were randomly divided into two groups (*n* = 6 per group): the vector stone model group and the sET‐GPX4 stone model group. The dRfxCas13d‐eIF4GI‐GPX4 plasmid was packaged into Adeno‐associated virus (AAV) vectors. The mice were anesthetized to expose the kidney, the ureter was blocked using a hemostatic clip, and then the established sET‐GPX4 AVV (1 × 10^11^ µg per mouse) was injected into the renal pelvis. The expression of GPX4 was measured after 4 weeks. Thereafter, the kidney stone mouse model was constructed by injecting 80 mg kg^−1^ glyoxylic acid (Sigma–Aldrich; Merck KGaA) i.p. every day for 14 days. Subsequently, the experimental animals were anesthetized by injecting pentobarbital (50 mg kg^−1^) i.p. 1 day after the last treatment and the kidney tissues and blood samples were collected. Then all animals were euthanatized using pentobarbital (100 mg kg^−1^) after surgery.

### Protein Extraction and Enzymatic Digestion

After experimental interventions, total protein from cells was obtained and stored at −80 °C. Cell samples were removed from −80 °C freezer and added to four times the volume of lysis buffer (8 m urea, 1% protease inhibitor), then sonicated. The mixture was centrifuged at 4 °C, 12 000 × *g* for 10 min and the supernatant was transferred to a new centrifuge tube. Protein concentration was determined using the BCA assay. Proteins from each sample were enzymatically digested with equal amounts of enzyme using a consistent volume of lysis buffer. The sample was adjusted to a consistent volume, to which was added 1 volume of pre‐cooled acetone, followed by vortexing, the addition of four volumes of pre‐cooled acetone, and incubation at −20 °C for 2 h. After centrifugation at 4500 × *g* for 5 min, the supernatant was discarded and the precipitate was washed twice with pre‐cooled acetone. The precipitate was dried and added to Triethylammonium bicarbonate (TEAB) to a final concentration of 200 mm. The sample was sonicated and then mixed with trypsin at a protein‐to‐protease ratio of 1:50 (m/m) and incubated overnight. After adding dithiothreitol to a final concentration of 5 mm and reducing the volume to 56 °C for 30 min, Indole acetic acid (IAA) was added to a final concentration of 11 mm, and the mixture was incubated at room temperature in the dark for 15 min.

### Liquid Chromatography‐Mass Spectrometry (LC‐MS) Analysis and Protein Annotation (for 4D‐LFQ Proteomics)

Peptides were dissolved in mobile phase A solution (0.1% formic acid, 2% acetonitrile in water) and separated using a NanoElute ultra‐high‐performance liquid chromatography system. The gradient was set as follows: 0–1 min, 2–5% B; 1–76 min, 5–27% B; 76–82 min, 27–35% B; 82–86 min, 35–85% B, and the flow rate was maintained at 300.00 nL min^−1^. The peptides were ionized in the capillary ion source and analyzed by trapped ion mobility spectrometry‐time of flight mass spectrometry. The ion source voltage was set to 2.0 kV, and both the parent ion and its secondary fragments were detected and analyzed using high‐resolution time of flight. The mass range of the secondary mass spectrometry was set to 100–1700. The data acquisition mode used the parallel accumulation‐serial fragmentation mode. After collecting one primary mass spectrum, 10 parallel accumulation‐serial fragmentation mode spectra were collected for the secondary spectrum with a charge state of 0–5, and the dynamic exclusion time for tandem mass spectrometry was set to 30 s to avoid repeated scanning of the parent ion. Maxquant v1.6.15.0 was used for searching the second‐level mass spectrometry data. The search parameters included Homo_sapiens_9606 database (20 366 sequences), a reverse database for calculating the false discovery rate, and common contaminant libraries to eliminate the impact of contaminant proteins. Enzyme digestion was set as Trypsin/P, with missed cleavage sites set to two, and the minimum peptide length set to seven amino acid residues. The maximum number of modifications per peptide was set to five. The first‐level parent ion mass error tolerance was set to 20.0 ppm for both the first search and main search, and the mass error tolerance for the second‐level fragment ions was set to 20.0 ppm. Carbamidomethylation of cysteine was set as a fixed modification, and the variable modifications were acetyl (protein N‐terminus) and oxidation. The quantification method was set to label‐free quantitation (LFQ), and the false discovery rate for protein and peptide‐spectrum matches (PSM) identification were both set to 1%.

### Analysis of Functional Enrichment

Enrichment analysis was performed to classify the proteins based on Gene Ontology (GO) annotation into three categories, namely biological process, cellular compartment, and molecular function. A two‐tailed Fisher's exact test was used to determine the enrichment of differentially expressed proteins against all identified proteins in each category. A GO annotation with a corrected *p*‐value < 0.05 was considered significant. Pathway analysis was conducted using the Encyclopedia of Genes and Genomes (KEGG) database to identify enriched pathways by applying a two‐tailed Fisher's exact test to assess the enrichment of differentially expressed proteins against all identified proteins. A pathway with a corrected *p*‐value < 0.05 was considered significant, and pathways were classified into hierarchical categories based on the KEGG website. Furthermore, the InterPro database was used to conduct protein domain analysis, and a two‐tailed Fisher's exact test was used to determine the enrichment of differentially expressed proteins against all identified proteins for each category. Protein domains with a corrected *p*‐value < 0.05 were considered significant.

### Plasmid Design and Construction

During the construction of the dual‐vector translation regulation system, the DNA sequence encoding dRfxCas13d nuclease was chemically synthesized, cloned into a plasmid containing the CMV promoter, and fused with the eIF4GI domain (pSP4GI, the full‐length eIF4GI cDNA clone DKFZp762O191Q3, accession no. AL120751) at the C‐terminus of the plasmid skeleton as a functional element. The nuclear entry tool places nuclear localization signals (NLS) at both ends of the fusion protein sequence. Cytoplasmic tools do not require fusion of NLS. The complementary DNA sequence of sgRNA was cloned into a plasmid containing the U6 promoter as a target for identifying the target mRNA. For the construction of the single‐vector translation regulation system, non‐essential tool components were removed, such as fluorescent labels and NLS, and the DNA sequence complementary to the fusion protein element and sgRNA was inserted into a consensus skeleton to form the plasmid dRfxCas13d‐eIF4GI‐sgRNA. Expression of the fusion protein dRfxCas13d‐eIF4GI and sgRNA were driven by the CMV promoter and U6 promoter, respectively. The complementary sequence design of the sgRNA was performed using the online design website https://cas13design.nygenome.org. The sgRNA design webpage corresponding to the appropriate species was begun with, the name of the target protein was entered, and then the corresponding sequence was selected with a higher score for the region where sgRNA was targeted. Plasmid mapping and sequence information are available in Figures S1–S11 and Tables S1–S3 (Supporting Information).

### Quantitative Real‐time Reverse Transcription PCR (qRT‐PCR)

Total RNA was isolated from cells and mouse kidney tissues using Trizol reagent (15 596 026, Thermo Fisher) according to the manufacturer's instructions as described previously. RNA (2 µg) was converted into cDNA using the Hifair III 1st Strand cDNA Synthesis Kit (gDNA digester plus; 11139ES60, Yeasen Biotechnology Co., Ltd., Shanghai, China). Real‐time PCR was performed using a LightCycler 480 (Roche Diagnostics, USA) with Hieff UNICON Universal Blue qPCR SYBR Green Master Mix (11184ES08, Yeasen Biotechnology). The relative expression level of indicated genes was compared with that of GAPDH and expression fold changes were calculated using 2^−△△Ct^ methods. Each qRT‐PCR reaction was performed in triplicate. The primers used were as follows:

GAPDH, 5′‐GTCTCCTCTGACTTCAACAGCG‐3′(forward) and 5′‐ACCACCCTGTTGCTGTAGCCAA‐3′(reverse); GPX4, 5′‐GTAAACTACACTCAGCTCGTCGA‐3′(forward) and 5′‐TTGATCTCTTCGTTACTCCCTGG‐3′ (reverse);

SIRT1, 5′‐TAGACACGCTGGAACAGGTTGC‐3′(forward) and 5′‐CTCCTCGTACAGCTTCACAGTC‐3′ (reverse);

NIX, 5′‐TGTGGAAATGCACACCAGCAGG‐3′ (forward) and 5′‐CTACTGGACCAGTCTGATACCC‐3′ (reverse).

### Western Blotting (WB)

After experimental interventions, total protein from cells and mouse kidney tissues was obtained by lysis in cold RIPA buffer (G2002, Servicebio Technology Co., Ltd., Wuhan, China). Total protein was quantified using BCA assays (PC0020, Solarbio Science & Technology Co., Ltd., Beijing, China), and 30 µg protein was separated using 10–12% SDS‐PAGE and then transferred to Immun‐Blot PVDF Membranes (1 620 177, BIO‐RAD, Hercules, CA, USA). Membranes were blocked in rapid blocking buffer (PS108P, Epizyme Biomedical Technology Co., Ltd., Shanghai, China) for 10 min, washed four times with Tris‐buffered saline containing Tween 20 (TBST), and then incubated with the following primary antibodies at 4 °C for 12 h: anti‐GPX4 (A1933, 1:1000, ABclonal Technology Co., Ltd., Wuhan, China), anti‐CD71 (A5865, 1:1000, ABclonal), anti‐ASCL4 (22401‐1‐AP, 1:4000, Proteintech Group, Inc., USA), anti‐SIRT1 (13161‐1‐AP, 1:2000, Proteintech), anti‐NIX (A24803, 1:1000, ABclonal), anti‐eIF4G1 (ab2609, 1:1000, Abcam), anti‐FTH1 (ab65080, 1:1000, Abcam), anti‐PCBP2 (A23987, 1:1000, ABclonal), anti‐PRNP (A21570, 1:1000, ABclonal), anti‐Tubulin (11224‐1‐AP, 1:10 000, Proteintech), anti‐GAPDH (10494‐1‐AP, 1:2,0000, Proteintech), and anti‐β actin (20536‐1‐AP, 1:4000, Proteintech). The membranes were then washed with TBST buffer as previously described and incubated with secondary antibodies for 1 h at room temperature. Finally, the relative expression levels of each protein were observed by Odyssey dual‐color infrared laser imager (LI COR, Lincoln, NB, USA) and ChemiDoc XRS system (BIO‐RAD, USA), and the gray values were analyzed using ImageJ software (ImageJ v1.51j8, NIH; Bethesda, MD, USA).

### RNA Immunoprecipitation

For the RNA immunoprecipitation analysis, a protocol adapted from Abcam was employed(https://www.abcam.com/epigenetics/rna‐immunoprecipitation‐rip‐protocol). HK‐2 cells were cultivated on 10 cm plates and subsequently transfected with the specified plasmids. Post‐transfection, the cells were exposed to ultraviolet radiation at a wavelength of 254 nm and an energy density of 300 mJ cm^−^^2^. Subsequent to irradiation, cytoplasmic, and nuclear fractions were separated the following day. Sonication was applied to the nuclear fractions for five intervals (alternating between 30 s on and 30 s off) utilizing the Bioruptor Pico apparatus. Overnight at 4 °C, the respective lysates underwent immunoprecipitation with antibodies targeting specific proteins. To capture these antibodies, Protein A/G magnetic beads supplied by Invitrogen were introduced, followed by a sequence of washes to eliminate non‐specifically bound antibodies. Using an anti‐GPX4 antibody, GPX4 was immunoprecipitated from both the nuclear and cytoplasmic extracts. To purify the RNA, lysates were treated with proteinase K and incubated at 55 °C overnight. The RNA was subsequently isolated via a chloroform‐based extraction process using materials from WAKO and Trizol reagent from Thermo Fisher Scientific, Germany. qRT‐PCR was then performed to quantify the RNA.

### mRNA Stability

HK‐2 cells underwent a 24‐h pre‐transfection process with vectors. Following this, the cells were treated with 5 µg mL^−1^ of actinomycin D (Act‐D, Catalog No. A9415, Sigma–Aldrich, St. Louis, MO, USA) for designated durations. Subsequently, the cells were harvested and RNA was extracted for analysis by qRT‐PCR. The half‐life of the mRNA (t1/2) was determined by applying the formula ln(2)/slope.

### Polysome Profiling

Sucrose density gradient centrifugation was employed to generate polysome profiles from HEK293T cells. Cells that had been transfected with specific plasmids were incubated with cycloheximide (CHX) at a concentration of 100 µg mL^−1^ (sourced from Biodee, China) for a duration of 10 min to arrest translation before cell disruption. Lysis was carried out using 150 µL of a buffer consisting of 10 mm HEPES at pH 7.4, 5 mm MgCl_2_, 150 mm KCl, 0.5% NP‐40, 100 µg mL^−1^ CHX, 1 mm dithiothreitol, along with 200 units each of RNase inhibitor and protease inhibitor cocktail without EDTA (RNase‐in from Tiangen, China and protease inhibitor from HX‐Bio, China, respectively). Lysates were then clarified by a 10 min centrifugation at 13 000 × *g* at a temperature of 4 °C. Following clarification, 300 µL of the supernatant was carefully loaded onto a pre‐formed linear sucrose gradient ranging from 20% to 45% (w/v), which was prepared in a solution containing 10 mm HEPES pH 7.4, 5 mm MgCl_2_, 150 mm KCl, 100 µg mL^−1^ CHX, and 1 mm dithiothreitol. The samples were ultracentrifuged at a force of 1 12 000 × *g* in a 4 °C environment for 2.5 h. After centrifugation, the gradients were displaced from the bottom and the absorbance at 260 nm was continuously recorded utilizing a Piston Gradient Fractionator (manufactured by BIOCOMP, Canada). Fractions corresponding to the 260 nm absorbance peaks were collected, and RNA was subsequently isolated using 1.5 mL of Trizol LS reagent according to the protocol provided by Invitrogen.

### Cell Count Kit‐8 (CCK‐8) Assay

Cells were seeded into 96‐well plates and subjected to their respective interventions. Cell viability was determined using the CCK‐8 assay (BS350B, Biosharp, China). After the intervention, 100 µL of the CCK‐8 solution (basal medium 90 µL + CCK‐8 10 µL) was added to each well, and the cells were incubated for 2 h at 37 °C. Absorbance was measured at 450 nm with a microplate reader (EnSight, PerkinElmer, USA).

### Measurement of T‐AOC, SOD, MDA, and LDH

Following interventions, the classic indicators T‐AOC, SOD, MDA, and LDH, were detected to measure the levels of antioxidant and oxidative stress. In the present study, the levels of T‐AOC, SOD, MDA, and LDH were measured using kits from Nanjing Jiancheng Bioengineering Institute, (China): T‐AOC assay kit (A015‐2‐1), SOD assay kit (A001‐3‐2), MDA assay kit (A003‐4‐1), and LDH assay kit (A020‐2‐2), according to the corresponding kit instructions. The OD value was measured using a microplate reader (EnSight).

### Iron Assay

The iron assay kit (I291, DOJINDO, Japan) was used to detect the level of intracellular Fe2+. After treatment, both mouse kidney tissues and HK‐2 cells were homogenized in an iron assay buffer. The samples and reagents were used in strict accordance with the kit instructions, and the absorbance was measured at 593 nm using a microplate reader (EnSight).

### Reactive Oxygen Species (ROS)

Intracellular ROS were detected using the ROS Assay Kit‐Highly Sensitive DCFH‐DA (R252, Dojindo Laboratories, Japan). Once DCFH‐DA enters the cytoplasm, it can be oxidized to produce green fluorescence. Following treatment, the medium was removed and the configured working solution was then added and incubated at 37 °C for 30 min in the dark. Following incubation, the working solution was removed, and the cells were washed twice with Hank's Balanced Salt Solution (containing Ca^2+^ and Mg^2+^). Finally, the fluorescence intensity was observed using an inverted fluorescence microscope (IX71, Olympus, Japan) at ×200 magnification.

### Lipid Peroxidation Assessed by Liperfluo Staining

The cells (15 000 cells per well) were seeded in 24‐well plates, grown for 24 h after seeding, and then treated with CaOx (2 mm) for 24 h. The degree of lipid peroxidation was assessed by Liperfluo (L248, Dojindo Laboratories). The processed cells were stained with Liperfluo (10 µm) for 60 min at 37 °C. Finally, the fluorescence intensity was observed using a flow cytometer (CytoFlex, Beckman Coulter, USA) and analyzed by FlowJo 10.6.2 software.

### Immunofluorescence Assay

The cells (15 000 cells per well) were seeded in 24‐well plates and treated as described above after growing to 70% confluence. Then the cells were washed three times with PBS. The cells were then fixed with 4% paraformaldehyde (P0099, Beyotime Biotech Inc., Shanghai, China) for 15 min and washed three times with PBS. The cells were blocked with 5% BSA (ST2249, Beyotime Biotech) at room temperature and washed three times with PBS. Then the cells were treated with anti‐GPX4 antibodies (A1933, 1:200, ABclonal Technology) at 4 °C for 12 h. Subsequently, the cells were washed with PBS as previously described and incubated with secondary antibodies (4412, Cell Signaling Technology) at room temperature for 1 h. Finally, the coverslips were blocked with the anti‐fluorescence quencher with DAPI (P0131, Beyotime Biotech). The fluorescence intensity was measured using a positive fluorescence microscope (BX53, Olympus, Japan) at x200 magnification.

### Histological Analysis

The expression of renal GPX4 was determined by immunohistochemical analysis. After treatment, the mouse kidneys were removed, fixed with 4% paraformaldehyde (Beyotime Biotech), and embedded in paraffin. The tissue paraffin blocks were sliced and then immersed in xylene for 30 min, followed by dewaxing in an ethanol gradient solution. After the repair antigen was performed, anti‐GPX4 (1:100) was added to the slices, and incubated at 4 °C for 12 h. Then the secondary antibody (5220–0336, SeraCare, USA) was added to the slices and incubated at room temperature for 1 h. After washing, developing the stain, counterstaining, dehydration, and sealing, the slices were observed using a positive fluorescence microscope (BX53).

### Silver Nitrate Staining

After slicing and dewaxing, the kidney tissues were washed with distilled water three times, and silver nitrate solution was then dropped onto the slices before ultraviolet irradiation for 10 min. Then, the slices were washed with distilled water, hyposulfite solution was added to the slices, and the slices were placed at room temperature for 2 min. Then, the slices were counterstained using hematoxylin and eosin with a HE staining solution (G1120, Solarbio Science & Technology Co., Ltd., Beijing, China) before being observed under a positive fluorescence microscope (BX53).

### Serum Creatinine (CRE) and Blood Urea Nitrogen (BUN) Analyses

Following treatment, venous blood samples from the mice were collected. The blood samples were analyzed using an automatic clinical chemistry analyzer to measure CRE and BUN levels.

### Transmission Electron Microscopy (TEM)

A morphological assay by TEM was conducted to observe the morphological alterations of mitochondria following standard operating procedures. In brief, HK‐2 cells and kidney tissues were fixed after their respective experimental treatments. Thereafter, the cells and kidney tissues were dehydrated in ethanol and acetone for 15 min. A 60 nm thick slice was mounted onto a copper grid (200 mesh), and images were taken using TEM (JEOL, Tokyo, Japan). The number of normal and abnormal mitochondria in each group was analyzed.

### Statistical Analysis

All data from the experiments are expressed as the means ± standard error of the mean of ≥3 independent experiments. All statistical analyses were performed using GraphPad Prism software version 8.0 (GraphPad Software, Inc., San Diego, CA, USA). Comparisons between two groups were analyzed using Student's *t*‐test and the differences among multiple groups were analyzed with one‐way analysis of variance (ANOVA) followed by Turkey's multiple‐comparison test. *p* values were calculated through at least three independent experiments and were presented as the mean ± SD. *p* < 0.05 was considered to indicate a statistically significant difference.

## Conflict of Interest

The authors declare no conflict of interest.

## Supporting information

Supporting Information

Supplemental Table 1

## Data Availability

The data that support the findings of this study are available on request from the corresponding author. The data are not publicly available due to privacy or ethical restrictions.
