# Supplementary material for: Development and Application of the CRISPR‐dcas13d‐eIF4G Translational Regulatory System to Inhibit Ferroptosis in Calcium Oxalate Crystal‐Induced Kidney Injury
Source: Adv Sci (Weinh). 2024 Feb 21;11(17):2309234. doi: 10.1002/advs.202309234 (PMC11077677; doi:10.1002/advs.202309234)
Supplement: Supplementary file 1 — Supporting Information [file ADVS-11-2309234-s001.pdf]

ADVANCED SCIENCE
Open Access

Supporting Information

for *Adv. Sci.*, DOI 10.1002/advs.202309234

Development and Application of the CRISPR-dcas13d-eIF4G Translational Regulatory System to Inhibit Ferroptosis in Calcium Oxalate Crystal-Induced Kidney Injury

Ziqi He, Chao Song, Sheng Li, Caitao Dong, Wenbiao Liao, Yunhe Xiong, Sixing Yang and Yuchen Liu**

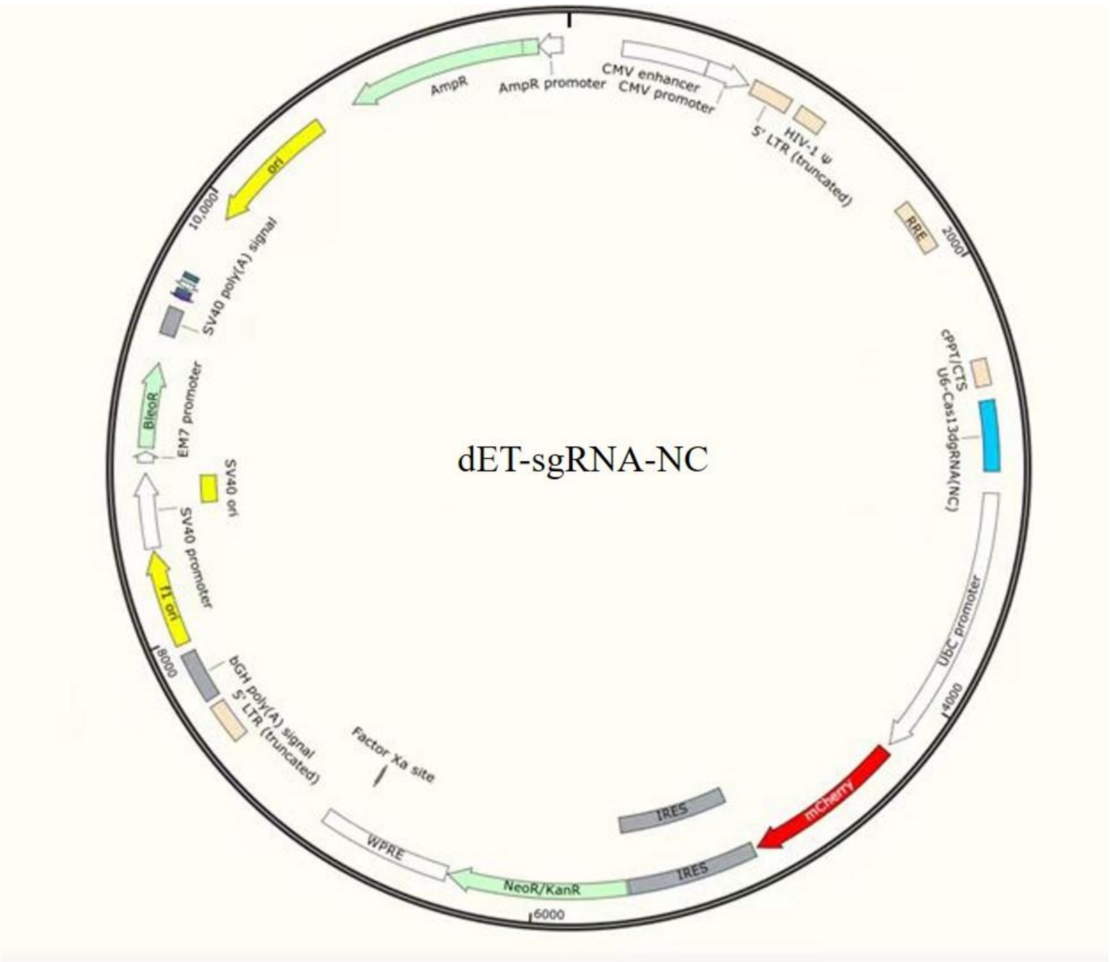

Figure S1 The map of the plasmid dET-sgRNA-NC.

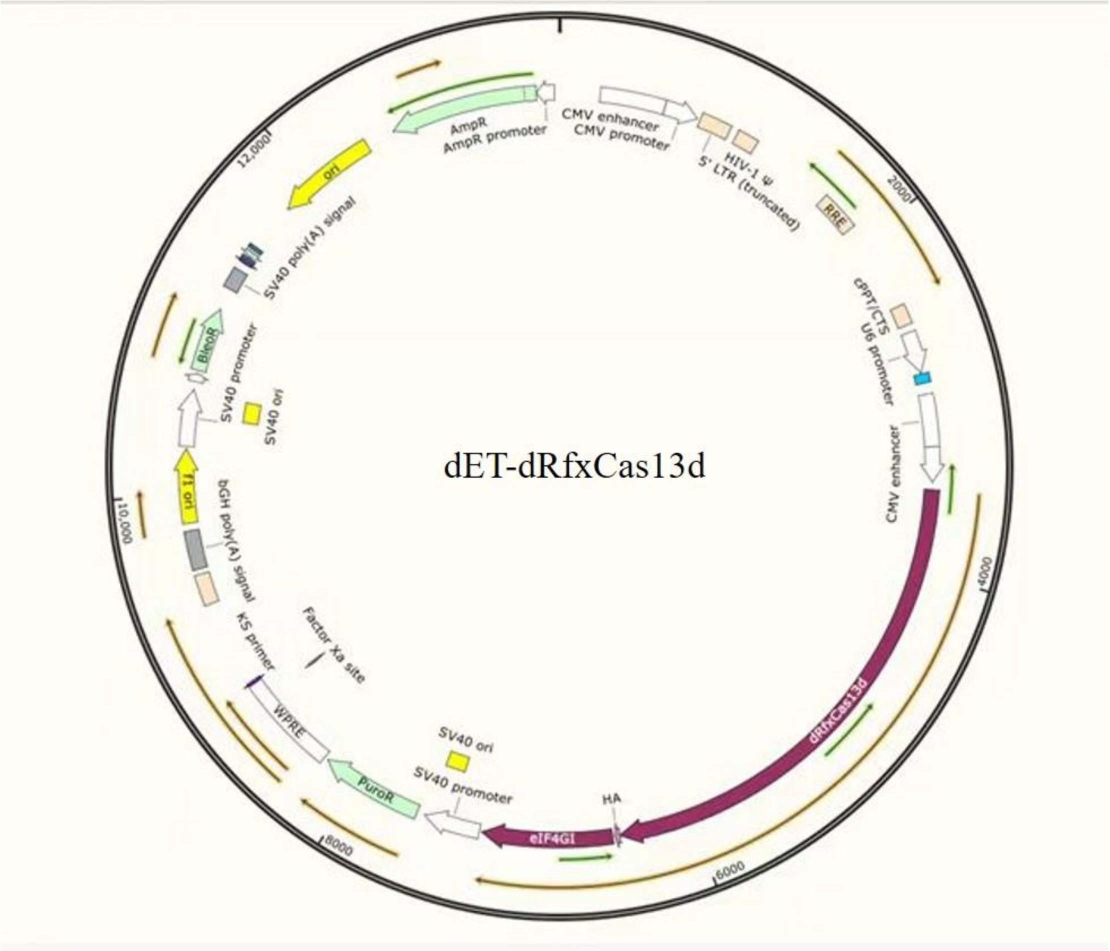

Figure S2 The map of the plasmid dET-dRfxCas13d.

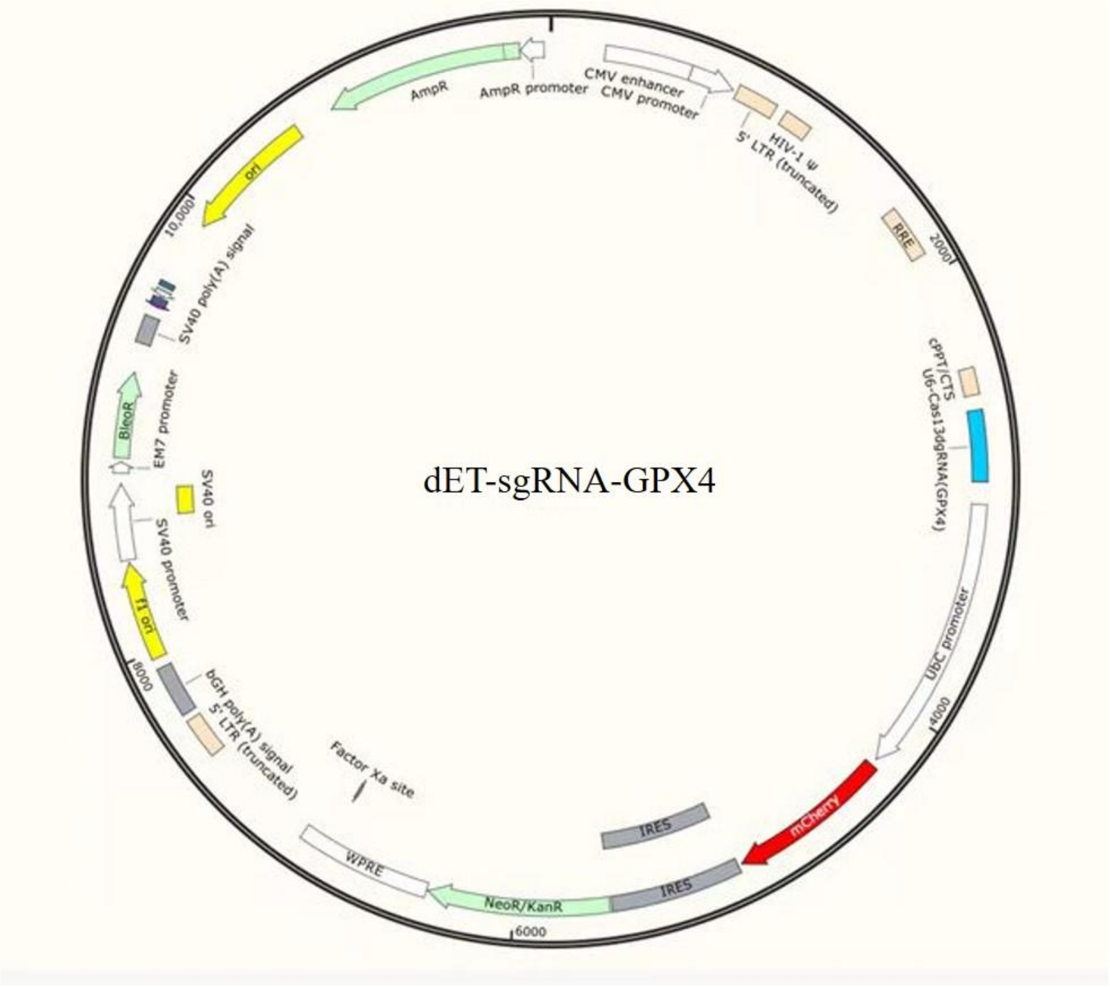

Figure S3 The map of the plasmid dET-sgRNA-GPX4.

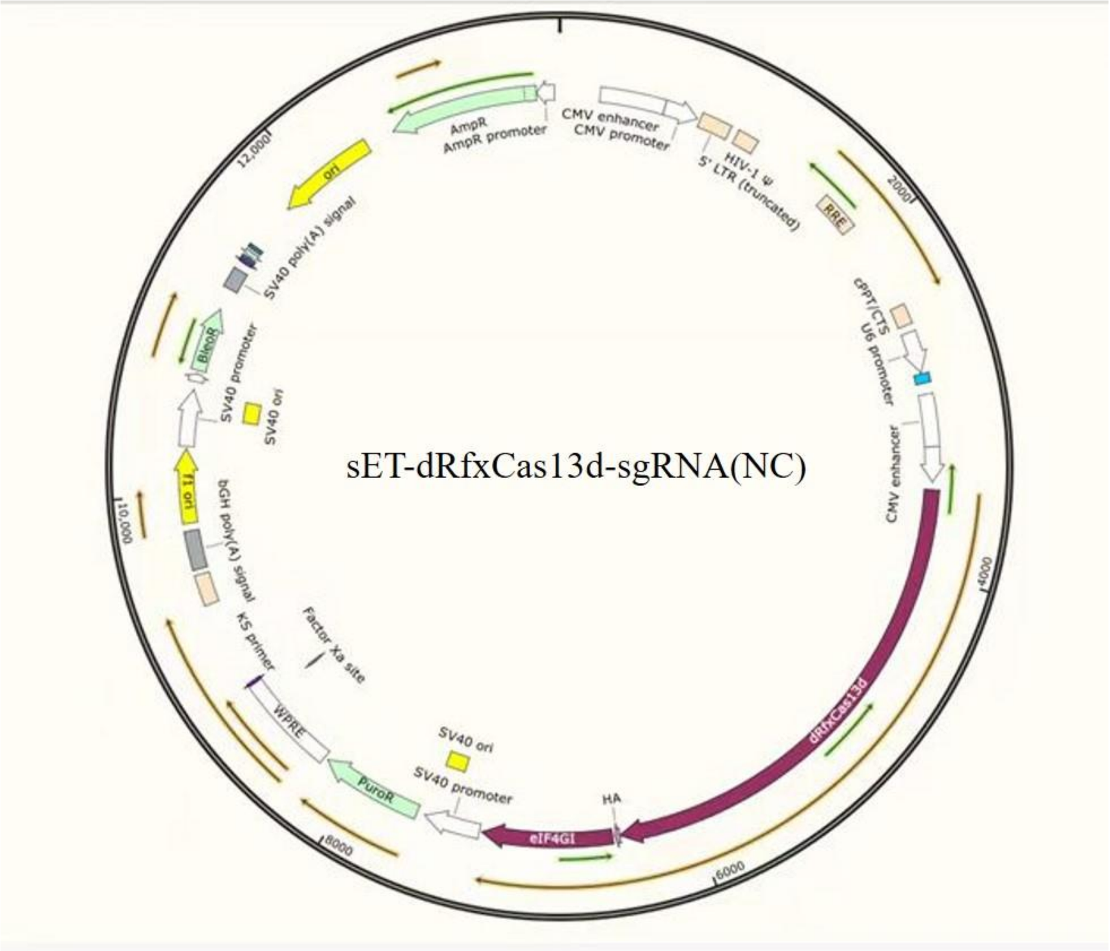

Figure S4 The map of the plasmid dET- dRfxCas13d-sgRNA(NC).

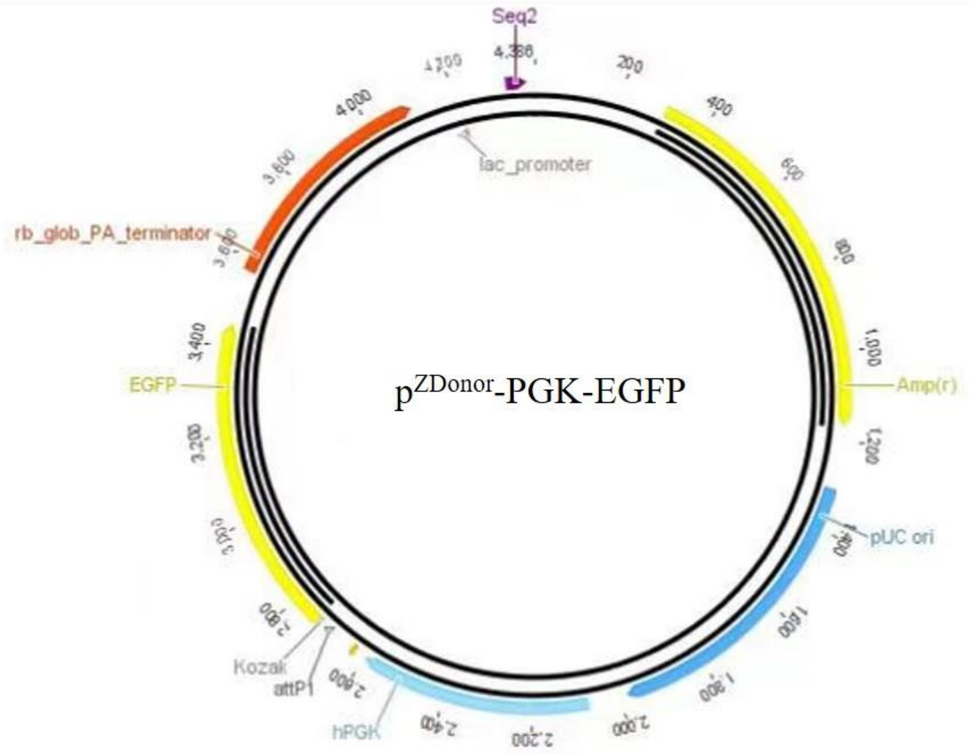

Figure S5 The map of the plasmid p^{ZDonor}-PGK-EGFP.

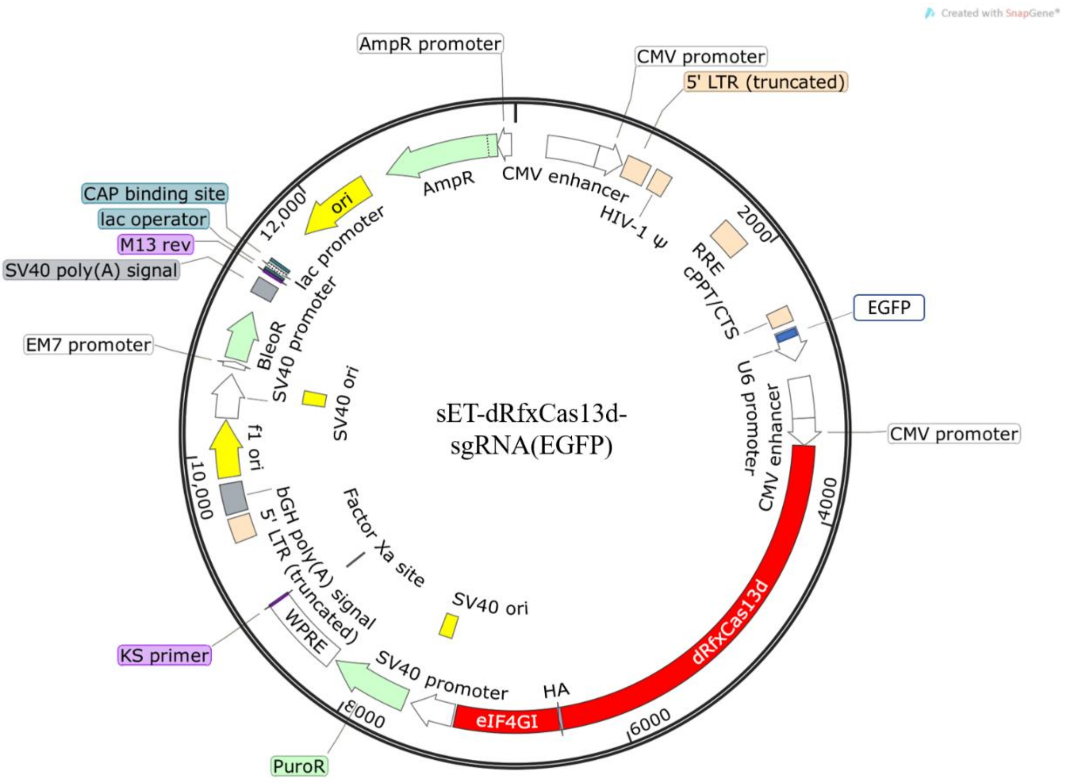

Figure S6 The map of the plasmid sET- dRfxCas13d-sgRNA (EGFP) and the plasmid was packaged into Lentiviral vectors.

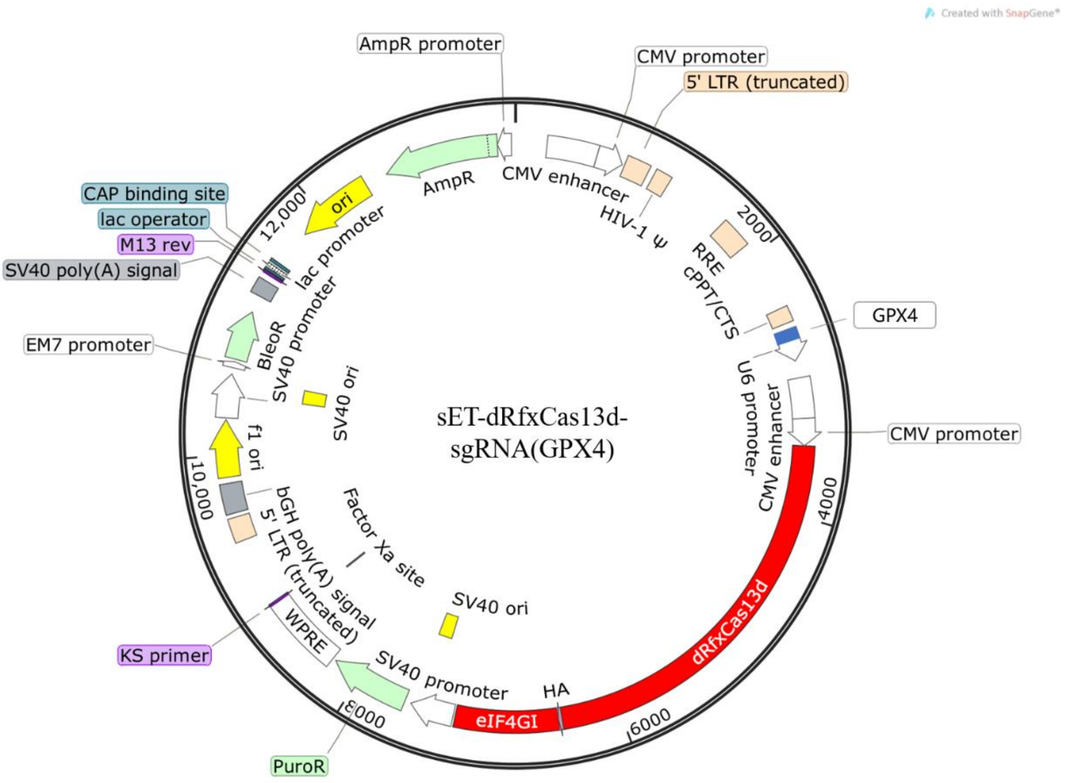

Figure S7 The map of the plasmid sET- dRfxCas13d-sgRNA (GPX4) and the plasmid was packaged into Lentiviral vectors.

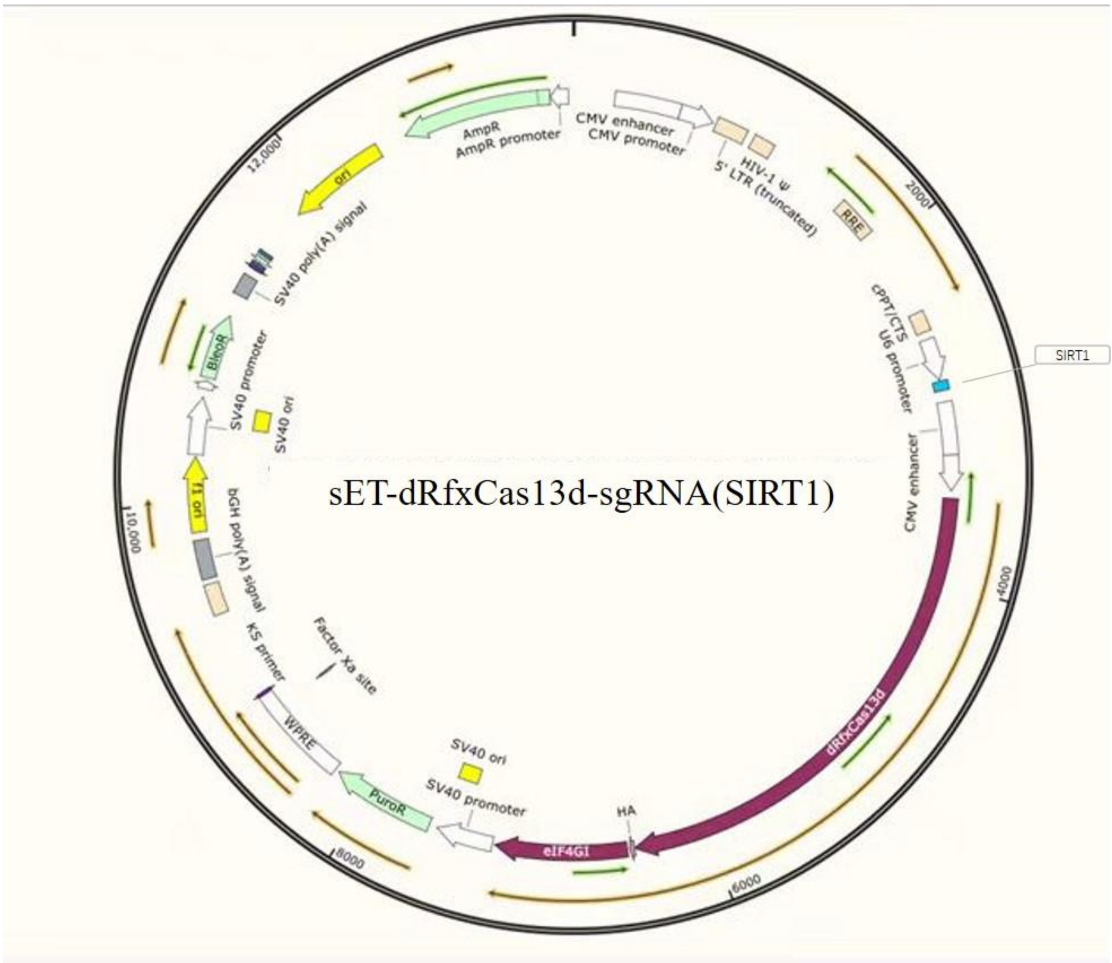

Figure S8 The map of the plasmid sET- dRfxCas13d-sgRNA (SIRT1) and the plasmid was packaged into Lentiviral vectors.

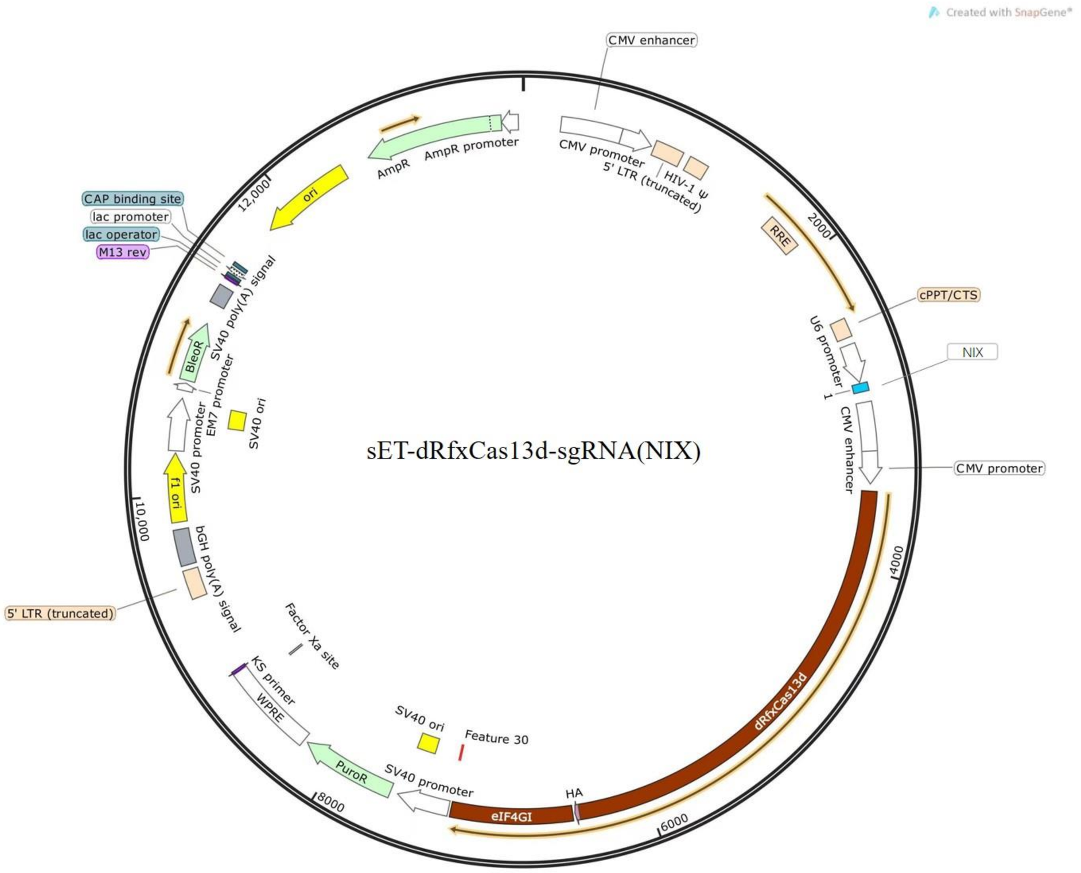

Figure S9 The map of the plasmid sET- dRfxCas13d-sgRNA (NIX) and the plasmid was packaged into Lentiviral vectors.

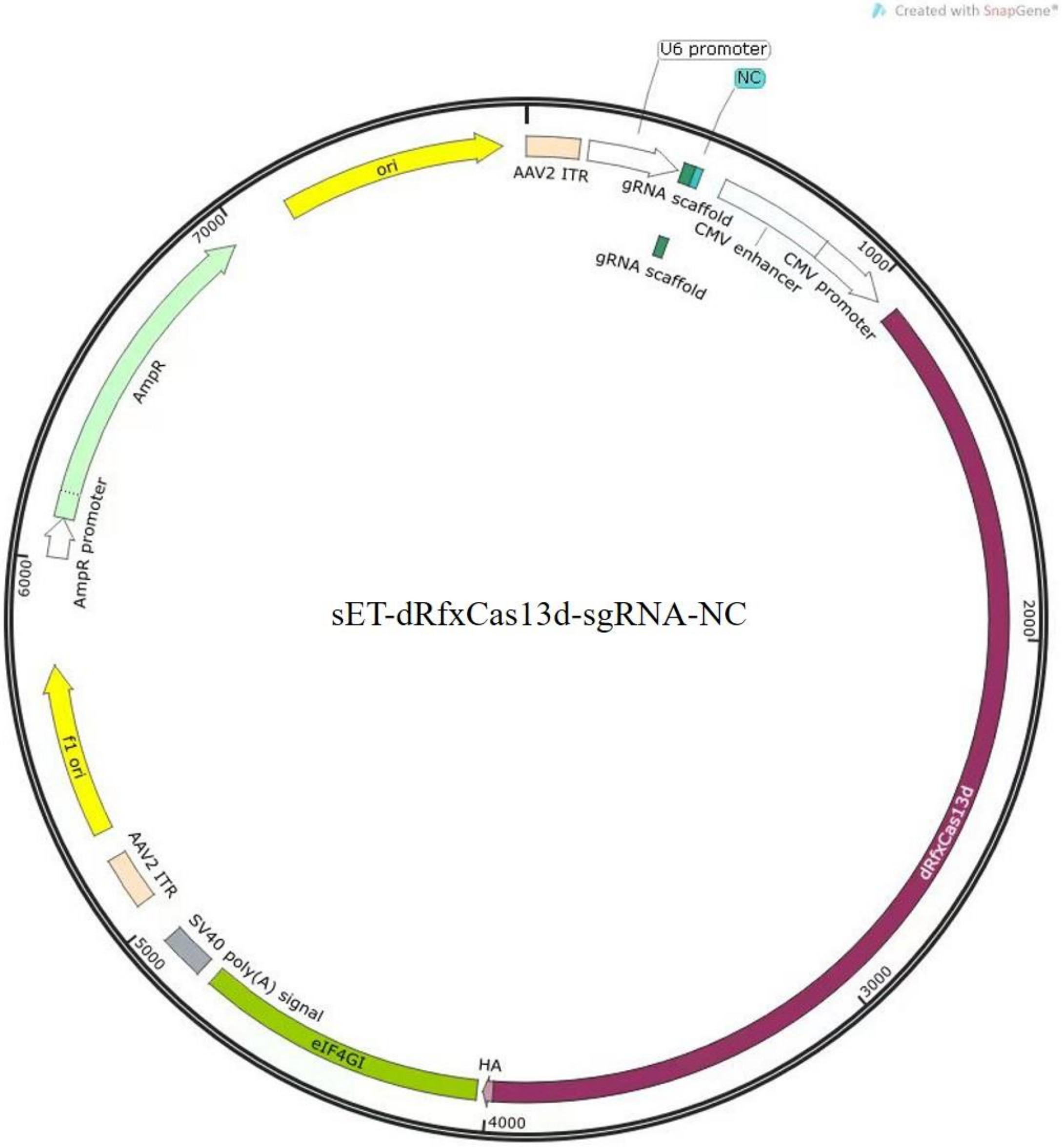

Figure S10 The map of the plasmid sET- dRfxCas13d-sgRNA (NC) and the plasmid was packaged into adeno-associated viral vectors.

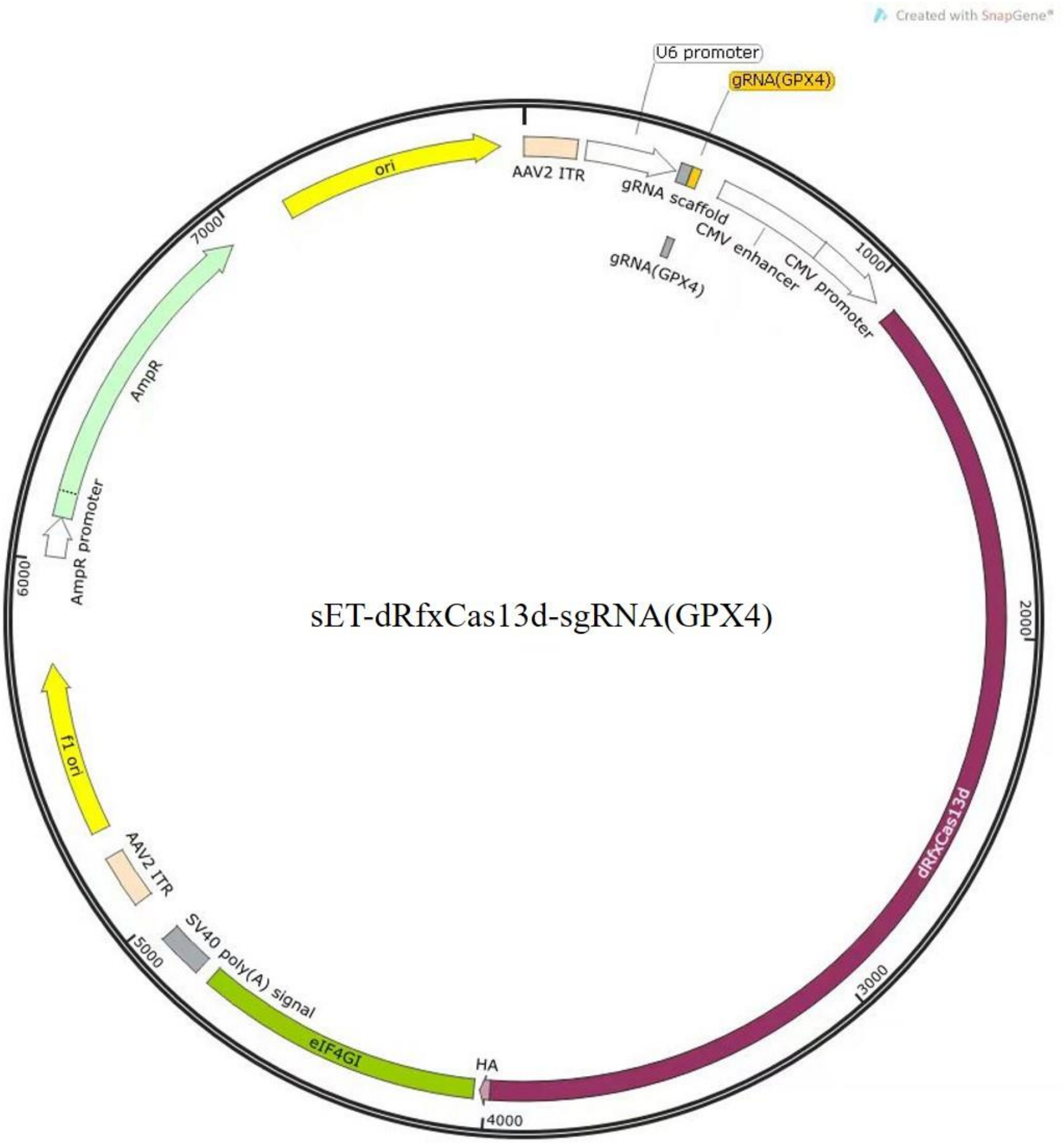

Figure S11 The map of the plasmid sET- dRfxCas13d-sgRNA (GPX4) and the plasmid was packaged into adeno-associated viral vectors.

A B

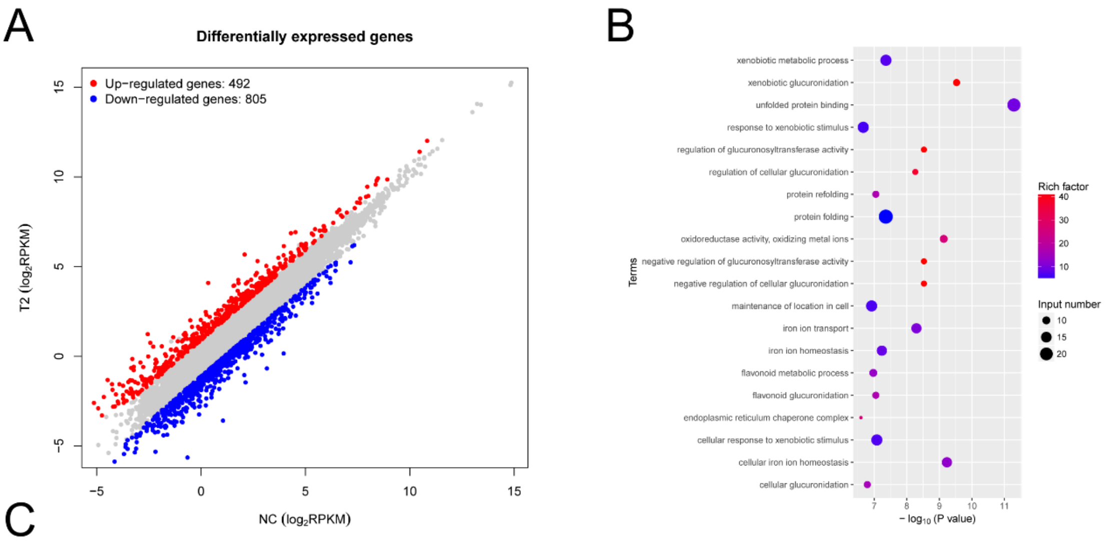

C

Protein accession	Gene name	NC_1	NC_2	NC_3	CaOx_1	CaOx_2	CaOx_3	CaOx_4	CaOx_5	CaOx_6
A6NCE7	MAP1LC3B2	0.782027	0.827117	0.850987	1.097777	1.03774	0.993848	1.146889	1.106768	1.156847
P02794	FTH1	1.282429	1.317133	1.27437	0.927912	0.883076	0.911611	0.81233	0.812147	0.778992
P04156	PRNP	0.868961	0.852382	0.885273	1.114055	1.065979	1.069423	1.044359	1.056962	1.042606
Q15366	PCBP2	1.006955	1.023518	1.0135	0.982934	0.990283	0.9935	0.984104	1.001873	1.003335
P36969	GPX4	1.137524	1.103058	1.113638	0.86741	0.873128	0.927952	0.99544	0.986196	0.995654
O60488	ACSL4	0.839217	0.795308	0.810469	1.108635	1.133592	1.127885	1.087128	1.056847	1.040919
P02786	TFRC	0.444696	0.415732	0.451442	1.126606	1.113339	1.090417	1.448987	1.488182	1.420598

D E

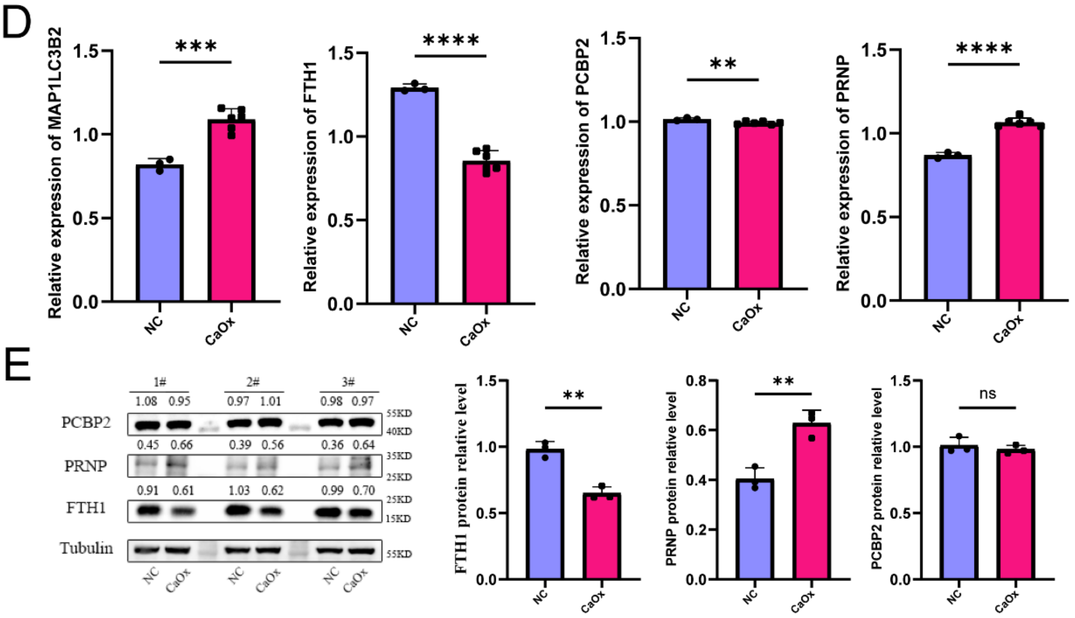

Figure S12 (A) The volcano plot for DEGs between CaOx crystal-induced cell injury models and normal controls. (B) GO term analysis of DEGs in pathway analysis. (C) All ferroptosis-related protein expression in HK-2 cells were determined by proteomic 4D-LFQ. (D) The protein (MAP1LC3B2, FTH1, PCBP2 and PRNP) expression in HK-2 cells were determined by proteomic 4D-LFQ. (E) Western blotting results show the protein expression levels of PCBP2, PRNP and FTH1 for two groups and bar graph shows the protein relative levels.

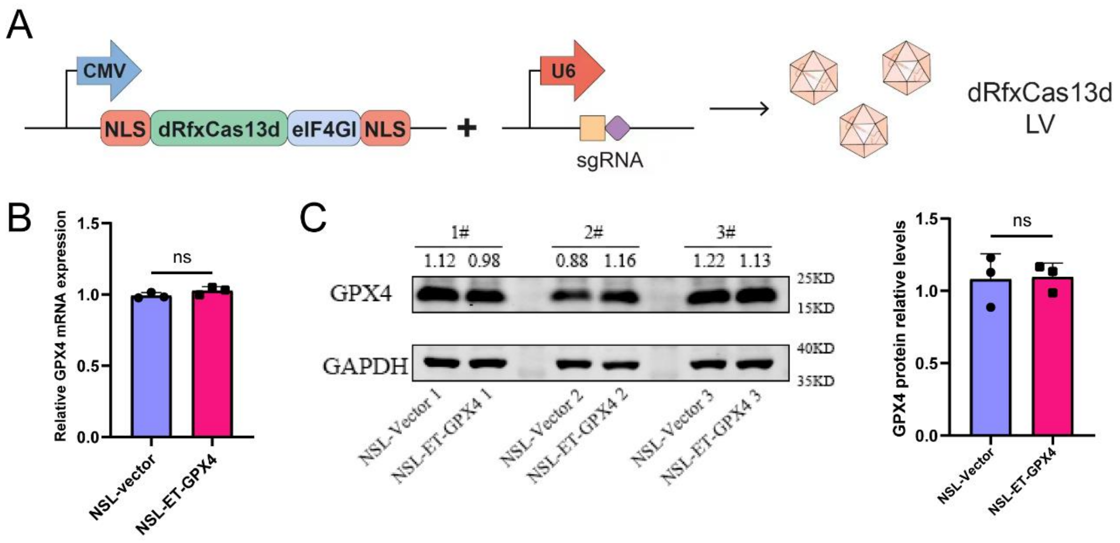

Figure S13 (A) The dRfxCas13d-eIF4G fusion protein, the NLS and sgRNA complementary DNA sequence of related genes were cloned into the plasmids containing CMV promoter and U6 promoter, respectively, for packaging into Lentiviral vectors. (B) The bar graph shows the mRNA level of GPX4. (C) Western blotting results show the protein expression levels of GPX4 for two groups and bar graph shows the protein relative levels.

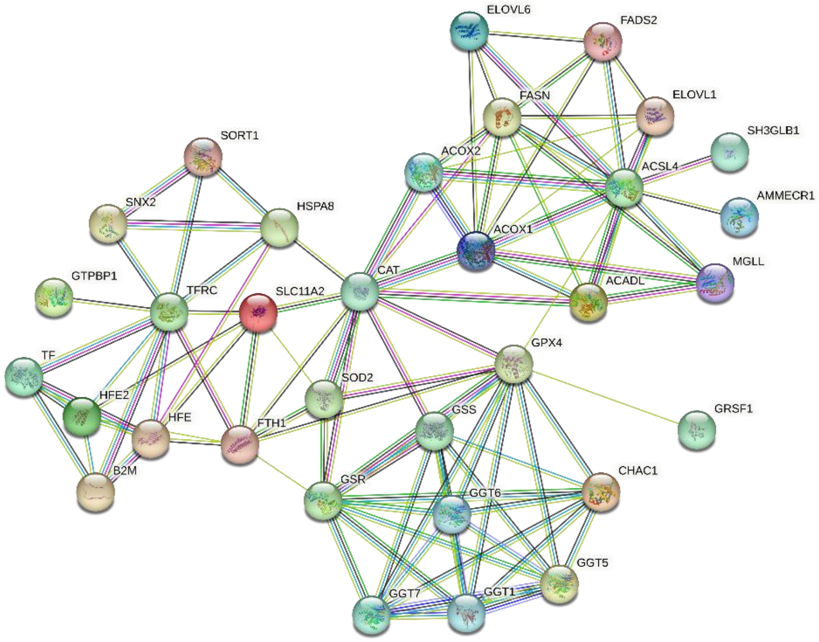

Figure S14 The protein-protein interaction (PPI) network of the selected DEPs.

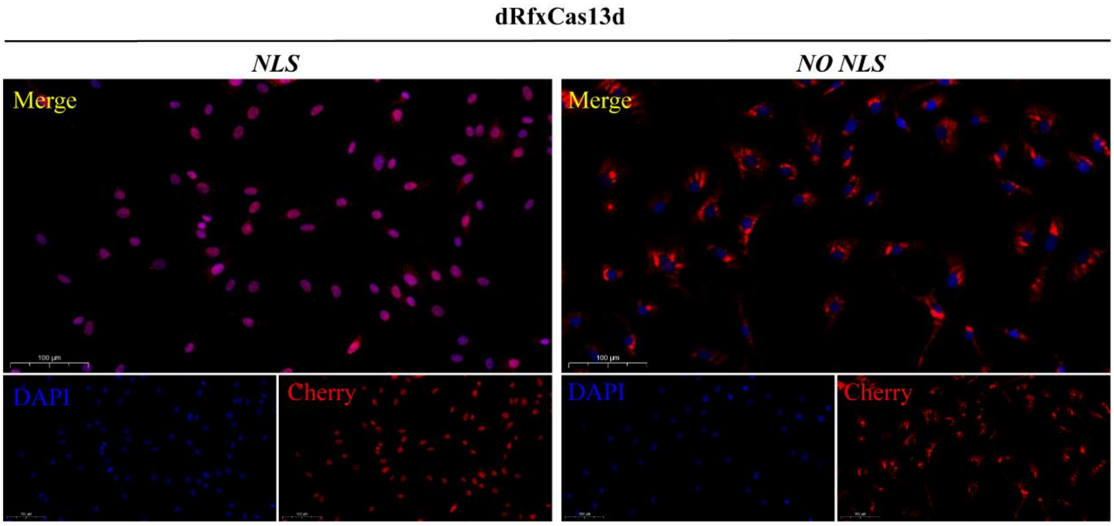

Figure S15 The subcellular localization of the single vector CRISPR-dRfxCas13d-eIF4GI translation enhancement tool. Immunofluorescence were performed to further determine the localization of Cherry fluorescent label (magnification, x200).

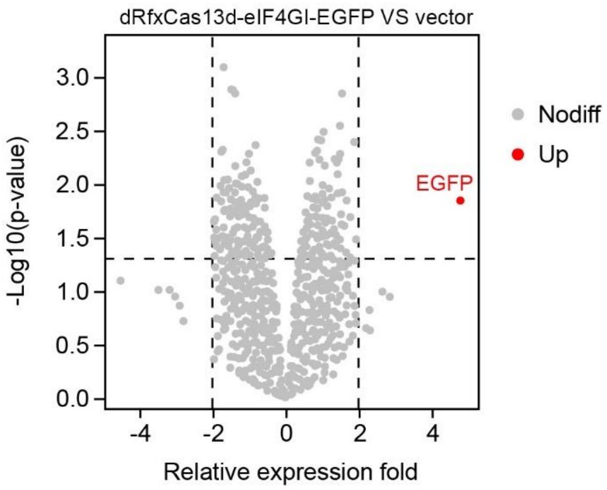

Figure S16 The volcano plot for DEGs between vector and sET-dRfxCas13d-sgRNA transferred with EGFP promotor.

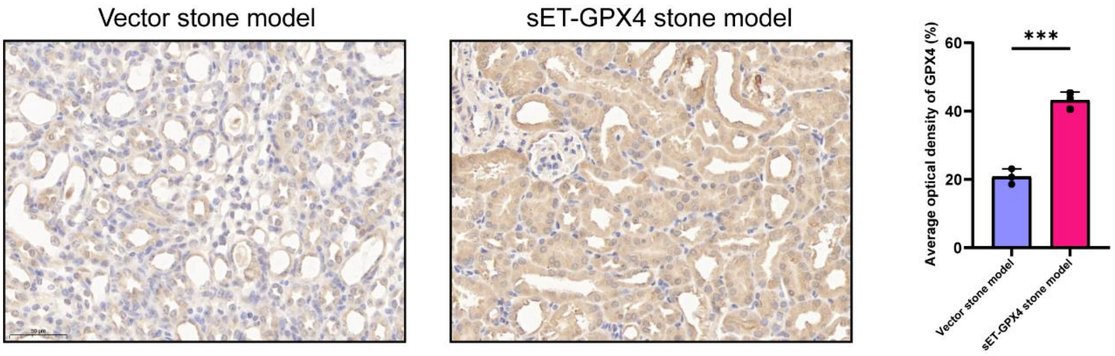

Figure S17 The images further show the degree of renal expression for GPX4 in two groups (magnification, x400) and bar graph shows relative protein levels.

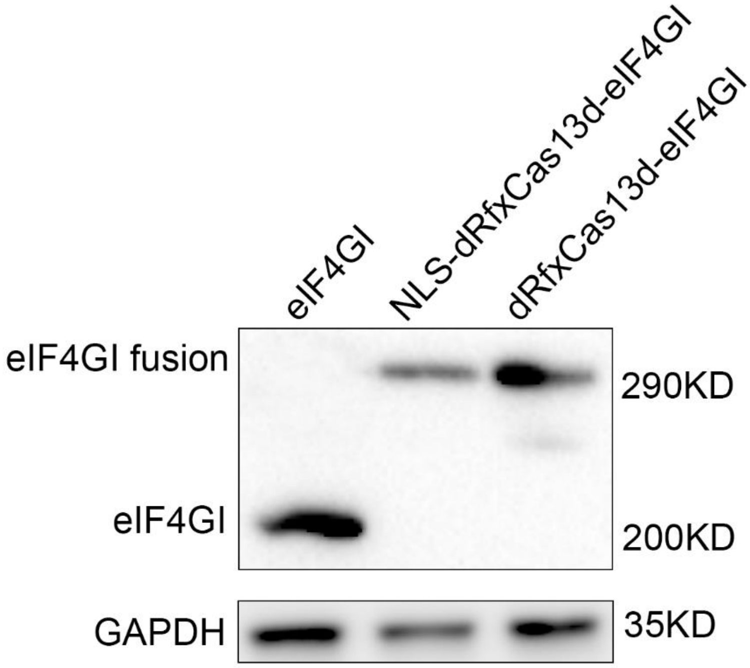

Figure S18 Expression of eIF4GI, NLS-dRfxCas13d-eIF4GI and dRfxCas13d-eIF4GI fusion protein in Hk-2 cells measured by western blotting using eIF4GI antibody.

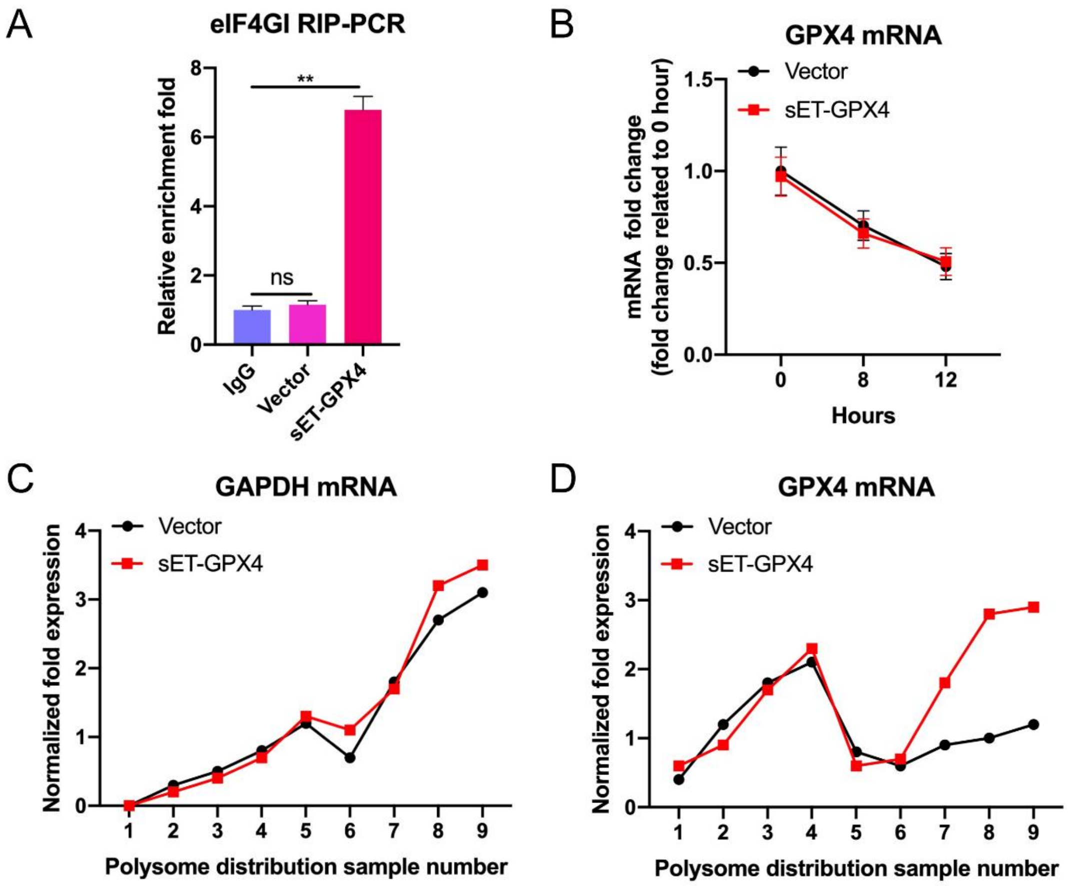

Figure S19 (A) HK-2 cells were pre-transfected as indicated for 24 h. Binding between eIF4GI and GPX4 mRNAs was checked by RIP-qPCR using eIF4GI antibody. (B) Actinomycin D (ActD) was added to HK-2 cell culture medium for 0, 8 or 12h to impair mRNA generation after vector and dET-GPX4 were transfected. qRT-PCR results showed that dET-GPX4 did not impair corresponding mRNA stability. (C) and (D) Polysome distribution of GPX4 mRNA in HK-2 cells, with GAPDH as internal control, with dET-GPX4 transfection or without transfection. This result confirmed that GPX4-targeted dRfxCas13d-eIF4G increased the number of ribosomes associated with the respective target mRNAs and thus enhanced their translation level.
